# Single-Cell Microgel Encapsulation Improves the Therapeutic Efficacy of Mesenchymal Stem Cells in Treating Intervertebral Disc Degeneration via Inhibiting Pyroptosis

**DOI:** 10.34133/research.0311

**Published:** 2024-02-16

**Authors:** Guanrui Huang, Haotian Shen, Kaiwang Xu, Yifan Shen, Jiale Jin, Guangyu Chu, Hongyuan Xing, Zhiyun Feng, Yue Wang

**Affiliations:** ^1^ Department of Orthopedic Surgery, The First Affiliated Hospital, Zhejiang University School of Medicine, Hangzhou 310003, China.; ^2^ Zhejiang University, Hangzhou 310058, China.

## Abstract

While mesenchymal stem cell (MSC) shows great potentials in treating intervertebral disc degeneration, most MSC die soon after intradiscal transplantation, resulting in inferior therapeutic efficacy. Currently, bulk hydrogels are the common solution to improve MSC survival in tissues, although hydrogel encapsulation impairs MSC migration and disrupts extracellular microenvironment. Cell hydrogel encapsulation has been proposed to overcome the limitation of traditional bulk hydrogels, yet this technique has not been used in treating disc degeneration. Using a layer-by-layer self-assembly technique, we fabricated alginate and gelatin microgel to encapsulate individual MSC for treating disc degeneration. The small size of microgel allowed intradiscal injection of coated MSC. We demonstrated that pyroptosis was involved in MSC death under oxidative stress stimulation, and microgel coating suppressed pyroptosis activation by maintaining mitochondria homeostasis. Microgel coating protected MSC in the harsh disc microenvironment, while retaining vital cellular functions such as migration, proliferation, and differentiation. In a rat model of disc degeneration, coated MSC exhibits prolonged retention in the disc and better efficacy of attenuating disc degeneration, as compared with bare MSC treatment alone. Further, microgel-coated MSC exhibited improved therapeutic effects in treating disc degeneration via suppressing the activation of pyroptosis in the disc. For the first time, microgel-encapsulated MSC was used to treat disc degeneration and obtain encouraging outcomes. The developed biocompatible single-cell hydrogel is an effective strategy to protect MSC and maintain cellular functions and may be an efficacious approach to improving the efficacy of MSC therapy in treating disc degeneration. The objective of this study is to improve the efficacy of cell therapy for treating disc degeneration using single-cell hydrogel encapsulation and further to understand related cytoprotective mechanisms.

## Introduction

Disc degeneration is the common pathology for a variety of degenerative lumbar spinal disorders [1]. Characterized by decreased number of resident cells and fibrosis of the nucleus pulposus (NP) tissues [2], disc degeneration is an aberrant, cell-mediated response to progressive structural failure [3]. The intervertebral disc has rather limited self-repair capability, and the microenvironment in a degenerated disc is harsh, with overexpressed inflammatory factors and accumulated reactive oxidative stress (ROS) [4]. Function recovery after disc degeneration thus is challenging [5]. Although currently there is no effective strategy to regenerate disc resident cells and reserve the catabolism of NP extracellular matrix (ECM) [6,7], evidence suggests that cell therapy may be a feasible approach to partially restoring the biological properties of the degenerated discs [8,9].

Mesenchymal stem cells (MSCs), which are nontumorigenic and easy to isolate [10], are commonly used seed cells in cell therapy [11,12]. Although reportedly intradiscal transplantation of MSCs can increase the deposition of proteoglycan and collagen or even improve radiological outcomes and pain relief [12,13], therapeutic efficacy of bare MSC virtually is limited [14,15]. The inferior efficacy of MSC therapy was attributed to the hypoxia and acidic microenvironment in degenerated discs, which is under high shear stress with complicated protease and cytokine networks [16]. In fact, when MSCs were injected to the disc, most of them died in a short time [17], and only a few survived [18]. Moreover, the high intradiscal pressure and narrowed space of the disc often restrict injection volume and lead to leakage [19]. In addition, the disc is largely avascular, which diminishes the use of intravenous delivery of MSCs [20]. Although repeated intradiscal injection can increase the number of MSCs delivered, needle puncture itself can exacerbate disc degeneration [21]. Protecting the transplanted MSCs and maintaining their physiological functions are crucial for improved therapeutic effects of MSCs in treating disc degeneration.

Evidence suggests that suppression of cell death can promote MSC retention. Although apoptosis [22,23] and necrosis [24,25] are the main approaches of MSC death, inhibition of apoptosis and necrosis only partially recover the viability of the transplanted MSCs [26,27]. It seems that other pathways may also involve in cell death. Pyroptosis, an acute cell death characterized by a cascade release of inflammatory factors from the dead cells [28], is a common pathology in various diseased tissues with ROS accumulation [29,30]. Pyroptotic cells have important roles in propagating in situ inflammation and trigger further pyroptosis of surrounding cells through the release of damage associated molecular patterns and inflammatory cytokines [31,32]. It has been proposed that pyroptosis may also occur in transplanted MSCs due to accumulated ROS in host tissues or gasdermins (GSDMs) released from local pyroptotic cells [33]. Pyroptosis-related cell death may further impair the curative effects of MSC therapy [34,35]. In theory, protecting MSCs against pyroptosis may improve the efficacy of MSC treatment.

Hydrogel encapsulation is a strategy used to create a protective shield for the transplanted cells [36,37]. Bulk hydrogels provide a stable bracket for transplanted or recruited stem cells [38,39] and, thus, can improve cell viability and enhance therapeutic effects of cell therapy. However, tiny voids of bulk hydrogels may hamper signal communications between the transplanted cells and host environment [40,41]. Moreover, the tight structure of crosslinked hydrogels hindered cell homing [42,43] and responsive cell migration to cytokine signals [44]. An ideal hydrogel for treating disc degeneration should mimic the advantageous features of the natural disc ECM, yet the low structure integrity of most hydrogels does not meet this requirement [45,46]. Biomaterials, which can protect stem cells and maintain cell independence, may promote therapeutic effect of MSCs in treating disc degeneration.

Microgel, an innovative design of hydrogel, was proposed to overcome limitations of the traditional bulk hydrogels [47–49]. Microgels are shells of biocompatible materials over cell surface [47], and the thin hydrogel layer does not alter cell sensitivity to tissue ECM and cell migration ability [47,48,50]. Previous studies have shown that single-cell hydrogel coating improved the regeneration of myocardium [51], cartilage [52], and bone [53], indicating that microgels may have potential in tissue engineering. However, methods for developing nanogels, such as electrostatic droplet extrusion [54] and microfluidic emulsion [53], are complicated and typically require specific equipment that may cause mechanical or chemical damages to cells [47]. By depositing layers of oppositely charged biocompatible materials, the layer-by-layer (LbL) self-assembly method is a relatively easy and efficient approach to constructing single-cell hydrogel encapsulation [55]. Importantly, this technique can minimize the damage to cells during encapsulation [48,56].

In this study, we adopted the LbL self-assembly method to encapsulate individual MSC and found that the cell surface nanogel can promote cell survival under oxidative stress. We found that microgel coating can effectively suppress pyroptosis activation and sustain mitochondria dysfunction. Moreover, microgel encapsulation improved the retention of MSCs in degenerated discs. In addition, we demonstrated that microgel improved the therapeutic efficacy of MSCs in a needle-puncture-induced model of disc degeneration. Findings suggested that microgel coating can improve the efficacy of MSCs in treating disc degeneration by attenuating pyroptosis.

## Results

### Microgel encapsulation protected individual MSC without altering cell structure and function

**Fig. 1.**
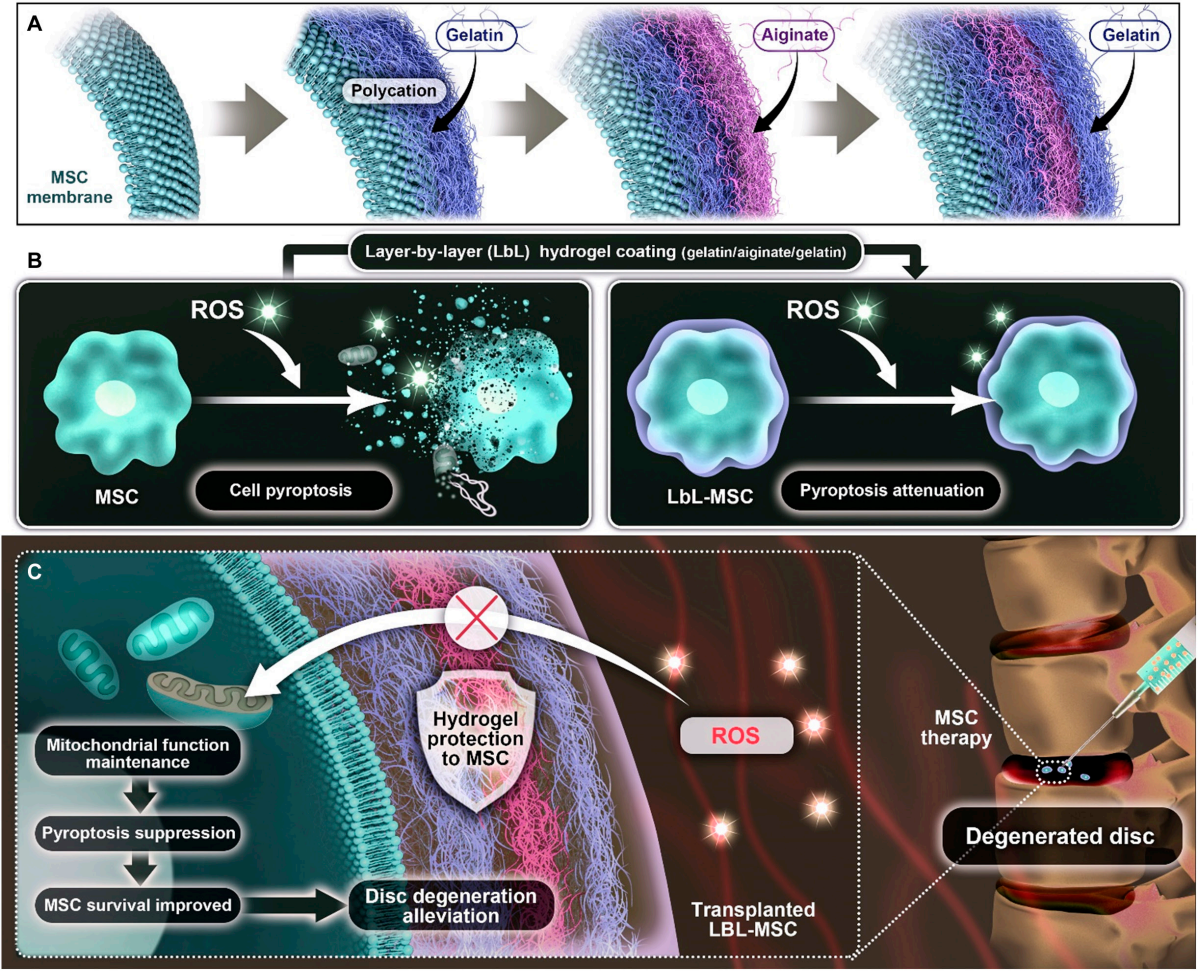
Schematic illustration of microgel coating as an improved method to enhance cell therapeutic efficacy to disc degeneration via inhibiting MSC pyroptosis. (A) MSC was encapsulated with trilayer of hydrogel coating individually through electric interaction. (B) Microgel coating attenuated pyroptosis activation in MSCs against ROS stimulation. (C) Mechanism of the microgel coating in improving MSC therapeutic efficacy to disc degeneration. Microgel alleviated ROS injury with mitochondria homeostasis maintenance. Mitochondria-dysfunction-induced pyroptosis was suppressed, and MSC survival was improved. As a result, LbL-MSCs showed improved effect on alleviating disc degeneration.

**Fig. 2.**
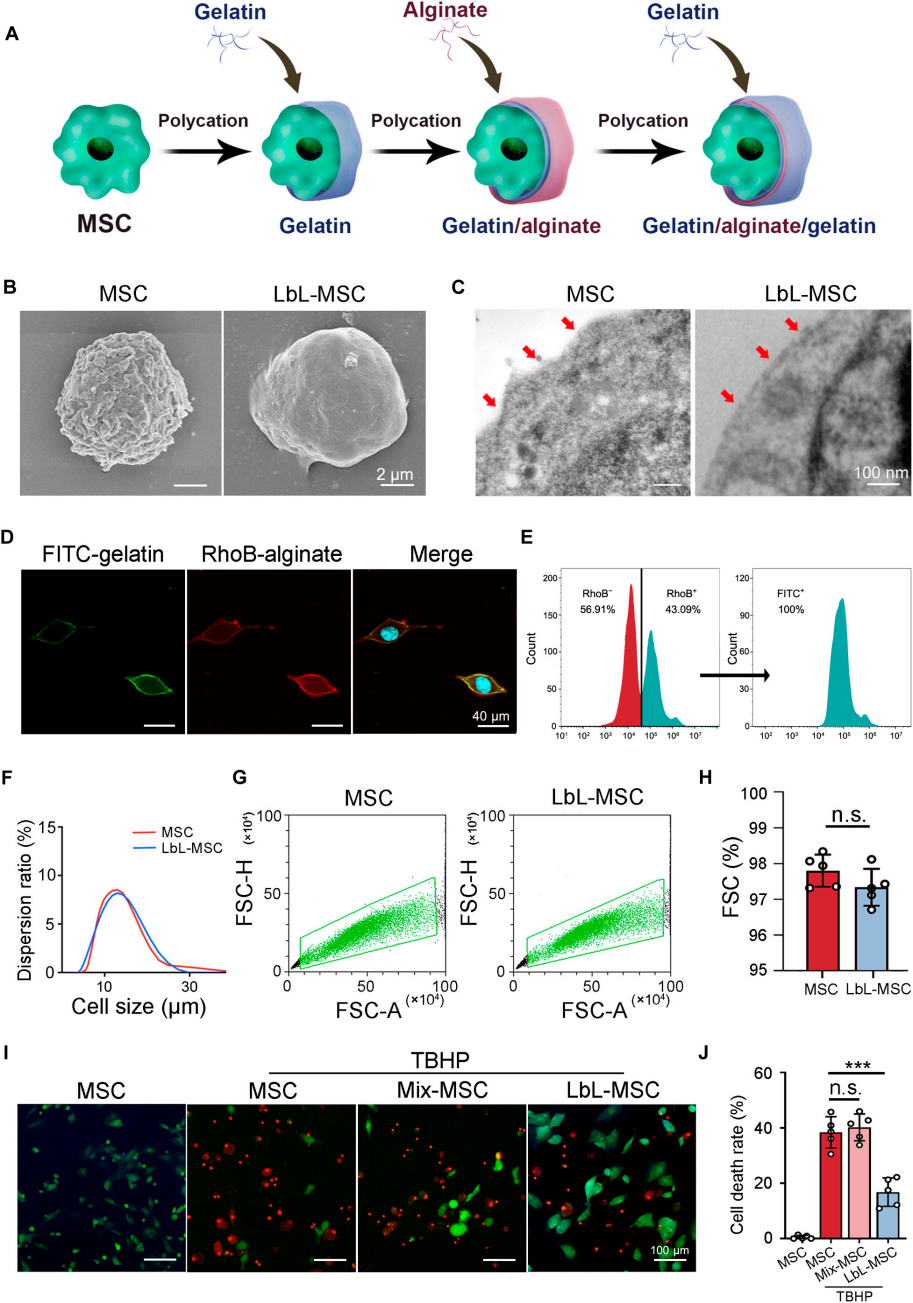
LbL self-assembly approach can efficiently generate microgel encapsulation of alginate and gelatin over the surface of individual MSC. (A) Schematic illustration of LbL encapsulation to achieve single-cell encapsulation by coating alginate and gelatin on cell surface. (B) Characterization of LbL encapsulation with SEM. Scale bars, 2 μm. (C) TEM demonstrated the modification of cell membrane surface by microgel encapsulation (red arrows, cell membrane). Scale bars, 100 nm. (D) LbL-MSCs under fluorescent microscope (blue, hoechst; green, FITC-labeled gelatin; red, RhoB-labeled alginate). Scale bars, 40 μm. (E) Cytometry analysis of the ratio of encapsulated cells. RhoB^+^ represented alginate encapsulation, and FITC^+^ represented gelatin encapsulation. (F) Size dispersion analysis of MSCs and LbL-MSCs. (G and H) Cell aggregation was detected and quantified with fluorescence-activated cell sorting. FSC-A, forward scatter area; FSC-H, forward scatter height. (I and J) Calcein-AM/PI staining revealed that LbL encapsulation protected cells under TBHP stimulation (green, calcein-AM, living cells; red, PI, dead cells). Scale bars, 100 μm. *n* = 5 independent experiments. n.s., not significant. ***P < 0.001.

An LbL self-assembly method, with 2 layers of gelatin and 1 layer of alginate, was used to encapsulate MSC (Figs. 1A and 2A). Scanning electron microscopy (SEM) revealed that a thin layer of hydrogel was formed over the surface of individual MSC (Fig. 2B and Fig. S1B). Different from the cloudy and fuzzy surface in bare MSC, transmission electron microscopy (TEM) demonstrated dense and distinct demarcations over the cell membrane of the encapsulated MSC, indicating that a layer of hydrogel was coated over the cell surface (Fig. 2C). Besides, encapsulation did not interrupt the adherent growth of the encapsulated cells (Fig. S1A).

To characterize the cell surface hydrogel, we fabricated fluorescein isothiocyanate (FITC)-labeled gelatin and rhodamine B (RhoB)-labeled alginate to encapsulate MSCs. FITC-gelatin and RhoB-alginate were colocalized on the surface of the cells (Fig. 2D). Flow cytometry analysis of RhoB showed 43.09% of cells were RhoB^+^ (representing cells encapsulated with alginate). All RhoB^+^ cells had high FITC fluorescence signal, suggesting successful dual encapsulation of MSCs. Hence, the overall dual-encapsulation ratio for MSCs was 43.09% (Fig. 2E). The mean diameters of MSCs and LbL-MSCs were not statistically different (13.84 μm versus 14.69 μm, *P* > 0.05; Fig. 2F), suggesting that encapsulation did not change cell size. Moreover, flow cytometry analysis demonstrated that minimal cell aggregation and cell independence were maintained after encapsulation (Fig. 2G and H). Overall, findings suggested that LbL self-assembly nanogel encapsulation did not alter cell structures, and cell integrity and individuality were preserved.

Impacts of encapsulation on the proliferation, migration, and differentiation of MSCs were then evaluated. Cell proliferation and migration abilities were not distinctively different between MSCs and LbL-MSCs in CCK-8 assays and transwell assays (Fig. S1C to E). Moreover, differentiation potentials of MSCs, including osteogenic, adipogenic, and chondrogenic potentials, remained unaltered after encapsulation (Fig. S1F). As ROS accumulated in degenerated discs may activate various cell death pathways and affect survival of transplanted cells, impact of oxidative stress on MSC and LbL-MSC was assessed. After TBHP incubation for 4 hours, more LbL-MSCs survived than MSCs (Fig. 2I; cell death rate 38% versus 18% for MSCs and LbL-MSCs, respectively, *P* < 0.001; Fig. 2J). Data supported that microgel encapsulation was able to protect MSC without altering cellular functions.

### Microgel coating improved the resistance of MSCs to oxidative stress by alleviating pyroptosis

To explore the mechanism underlying the protective function of LbL single-cell hydrogel encapsulation on MSCs, we assessed the transcriptomes of MSCs and LbL-MSCs under oxidative stress. Differential gene expression analysis with DESeq showed that the expression of cell death execution genes (*GSDMD, GSDME*, *MLKL*, and *BAX*) was significantly down-regulated in LbL-MSCs (Fig. S2A), suggesting that apoptosis, necrosis, and pyroptosis may be suppressed after coating. Results highlighted that microgel coating may take a role in the regulation of cell death.

**Fig. 3.**
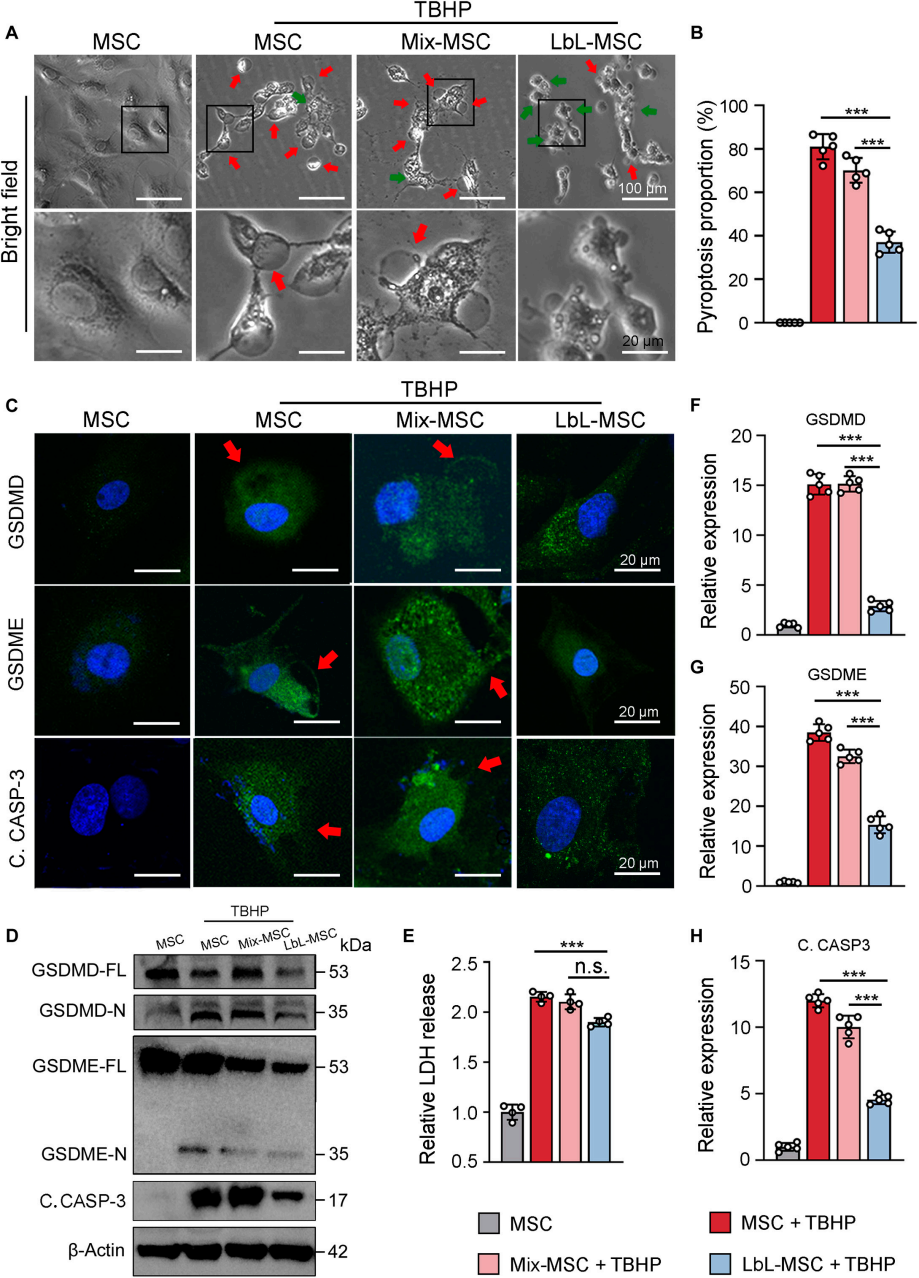
Microgel coating on cell surface protected MSCs from ROS-induced pyroptosis. (A) Pyroptotic morphology of MSCs and LbL-MSCs after TBHP treatment (red arrow, cells with pyroptosis morphology; green arrow, cells with nonpyroptosis morphology). Scale bars, 100 μm (top) and 20 μm (bottom). (B) Proportion of cells in pyroptosis morphology. (C) Immunofluorescence analysis of pyroptosis-related protein expression in MSCs and TBHP-treated MSCs and LbL-MSCs (red arrow, pyroptosis bubble). C. CASP-3, cleaved caspase-3. (D) Western blotting for pyroptosis-related proteins. (E) Relative LDH activity in the supernatant of normal and TBHP-treated cells. *n* = 4 independent experiments. (F to H) Quantification of pyroptosis-related protein. *n* = 5 independent experiments. ***P < 0.001.

To determine the key cell death pathway regulated by microgel coating, we further examined the morphology of MSCs after TBHP treatment. Under light microscope, characteristic morphology of pyroptosis activation, such as plasma membrane rupturing and blebbing (Fig. 3A), was commonly observed in TBHP-treated MSCs. After TBHP treatment, pyroptotic morphology was less common in LbL-MSCs than in MSCs, suggesting a cytoprotective effect of microgel against proptosis (Fig. 3B). TEM revealed marked cell membrane protruding and cell nucleus atrophy in MSC after TBHP treatment, while cell morphology remained unchanged in LbL-MSC and untreated MSC (Fig. S2C). These findings suggested that pyroptosis may be the key pathway regulated by microgel coating under ROS stimulation.

Next, effect of microgel on ROS-induced pyroptosis was further assessed. Released lactate dehydrogenases (LDHs) and interleukin-1β (IL-1β) were up-regulated after TBHP treatment in MSCs and attenuated in LbL-MSCs, suggesting that microgel suppressed cytoplasm content leakage (Fig. 3E and Fig. S2C). Both immunofluorescence analysis (Fig. 3C) and Western blotting (Fig. 3D) demonstrated that the expression of pyroptosis-related proteins, including cleaved caspase-3, activated GSDMD and GSDME (GSDMD-N and GSDME-N), was up-regulated in TBHP-treated MSCs (Fig. 3D and F to H and Fig. S2D to F) but can be attenuated after encapsulation. Results indicated that microgel encapsulation can alleviate oxidative-stress-induced pyroptosis.

### Microgel coating suppressed pyroptosis by sustaining mitochondria homeostasis

Upon the cytoprotective ability of microgel against pyroptosis, we further investigated the underlying mechanism. Gene Ontology enrichment analysis of TBHP-treated MSCs and LbL-MSCs showed a high fold enrichment for cellular response to DNA damage stimulus and response to oxidative stress (Fig. S3A). Further Gene Ontology enrichment in cellular components indicated the difference between MSCs and LbL-MSCs mainly located in mitochondria (Fig. S3B). As exposure to ROS can lead to loss of mitochondrial membrane potential and leakage of mitochondrial DNA (mtDNA) [57], we explored TBHP-induced alteration in mitochondria homeostasis and function.

**Fig. 4.**
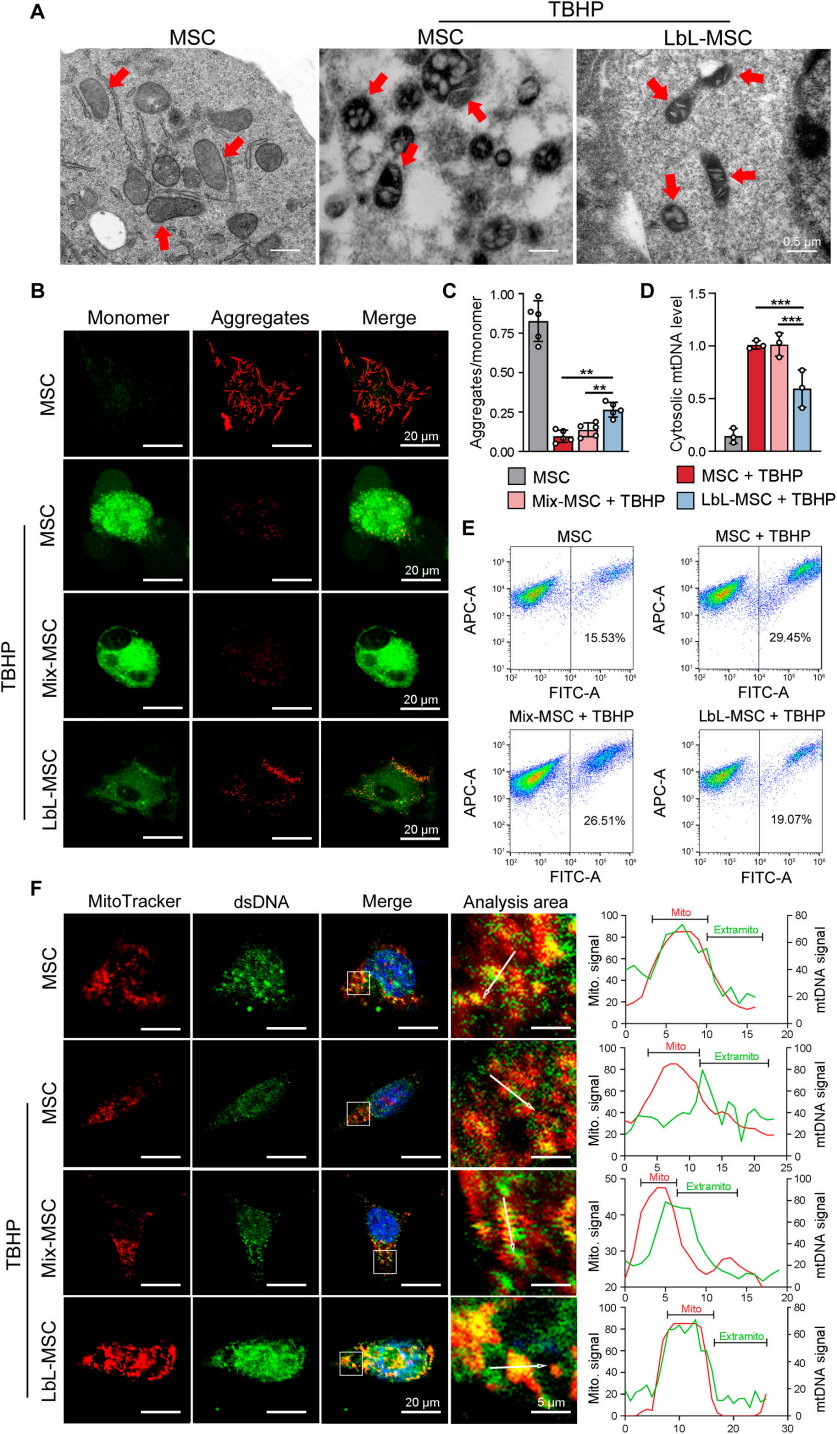
Microgel encapsulation protected MSCs from ROS-induced mitochondria dysfunction. (A) Detection of mitochondria morphology in different groups via TEM (red arrows, mitochondria). Scale bars, 0.5 μm. (B and C) Membrane potential of mitochondria was detected and quantified by JC-1 probe under fluorescence microscope. *n* = 5 independent experiments. (D) Quantitative polymerase chain reaction analysis of cytosolic mtDNA level in different treatment groups. *n* = 3 independent experiments. (E) Flow cytometry of JC-1 probe fluorescence status to reflect the mitochondrial membrane potential. APC, allophycocyanin. (F) Colocalization analysis of mitochondrial dsDNA signal and MitoTracker probe signal (red, mitochondria; green, mitochondria DNA). Scale bars, 20 and 5 μm. **P < 0.01; ***P < 0.001.

TEM revealed that mitochondria in MSCs were swollen and the double-layer membrane structure was damaged after TBHP treatment, while mitochondria in LbL-MSCs were slight swelled and showed clear cristae structure (Fig. 4A). JC-1 probe was used to detect the membrane potential shift and assess the mitochondrial function under oxidative stress (Fig. 4B). After treated with TBHP, the ratio of aggregates to monomers was higher in LbL-MSCs than in MSCs and MSCs mixed with hydrogel (0.27, 0.10, and 0.14, respectively; *P* < 0.01), indicating that microgel preserved mitochondria membrane potential (Fig. 4C). Flow cytometry analysis showed less cells with damaged mitochondria in LbL-MSCs than in MSCs and Mix-MSCs (MSCs mixed with hydrogel components without encapsulation) under oxidative stress (19.07%, 29.45%, and 26.51%, respectively; Fig. 4E). Data suggested that mitochondria membrane potential was decreased after ROS stimulation, while microgel coating protected mitochondria homeostasis.

In addition, lower level of mtDNA in cytoplasma was detected in TBHP-treated LbL-MSCs, as compared with TBHP-treated MSCs and Mix-MSCs (*P* < 0.001; Fig. 3D), suggesting that microgel can reduce the leakage of mtDNA out of mitochondria. To further assess mtDNA leakage, double-strand DNA (dsDNA) was labeled with immunofluorescence and mitochondria with MitoTracker probe. The signal peaks of plasma dsDNA and mitochondria overlapped spatially in normal MSCs and TBHP-treated LbL-MSCs, while separated in MSCs and Mix-MSCs after TBHP treatment (Fig. 4F). Microgel coating sustained mitochondria homeostasis and mitigated mtDNA leakage under oxidative stress, indicating that microgel can improve cell viability via maintaining mitochondria function and homeostasis.

While mtDNA leakage may induce various types of programmed cell death and participate in the progression of inflammation, its role in pyroptosis remained unclear. Therefore, we investigated whether mitochondria homeostasis was the underlying mechanism for microgel protection against pyroptosis. Carbonylcyanide-3-chlorophenylhydrazone (CCCP) was used to disrupt the mitochondrial membrane potential in MSCs and LbL-MSCs, and ciclosporin A (CsA) as a mitochondrial protector against the loss of mitochondria homeostasis. Both Western blotting (Fig. S3C and F) and immunofluorescence (Fig. S3D and G) analyses revealed pyroptosis activation following CCCP treatment, with up-regulated expression of GSDMD-N, GSDME-N, and cleaved caspase-3. CsA treatment alleviated CCCP-induced pyroptosis in MSCs. In LbL-MSCs, relative expression of GSDMD-N, GSDME-N, and cleaved caspase-3 was significantly lower, indicating that microgel attenuated pyroptosis activation. Besides, CCCP-induced LDH release was also reduced after cell surface hydrogel modification (Fig. S3E). Results indicated that under oxidative stress, mitochondria dysfunction activated pyroptosis, while microgel suppressed pyroptosis by sustaining mitochondria homeostasis.

### Microgel coating prolonged the retention of MSCs in degenerated discs

**Fig. 5.**
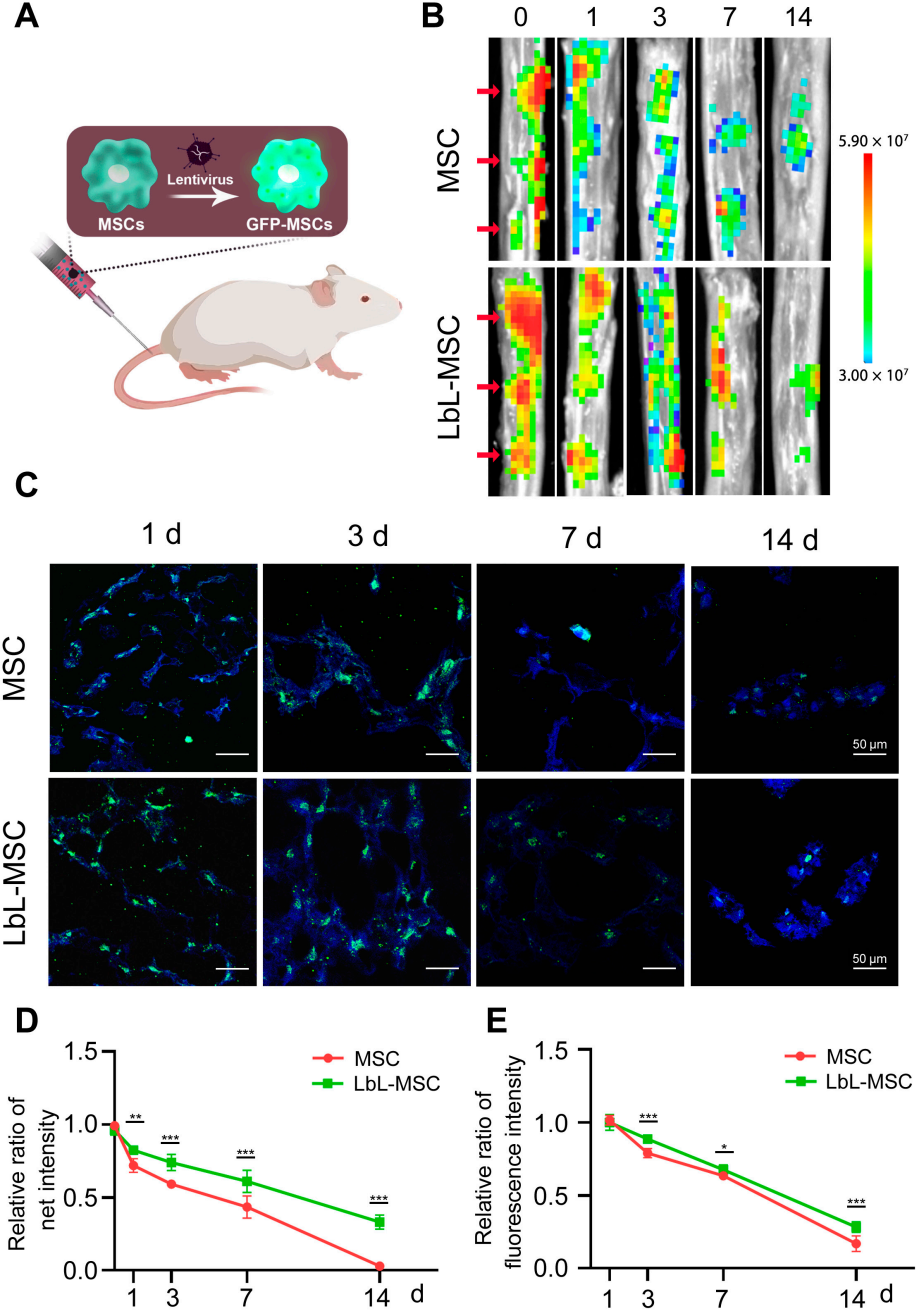
Single-cell microgel encapsulation prolonged the retention of MSCs in degenerated discs. (A) Schematic illustration of tracking transplanted MSCs in vivo. (B) In vivo optical imaging of GFP fluorescence in discs at various time points (red arrows, MSC or LbL-MSC implanted discs). (C) Immunofluorescence staining of transfected GFP fluorescence to detect injected cells. Scale bars, 50 μm. (D) Optical imaging measurement of signal for discs injected with MSCs and LbL-MSCs over various time points. (E) Proportion of fluorescence intensity signal, as from the reserved MSCs in the discs. *n* = 3 independent experiments. *P < 0.05; **P < 0.01; ***P < 0.001.

In light of the promising cytoprotective effect of microgel in vitro, we sought to investigate whether the single-cell hydrogel encapsulation can enhance the survival of transplanted MSCs in degenerated discs. First, a needle-puncture-induced disc degeneration model was established in rats. MSCs were engineered to express green fluorescent protein (GFP) and then injected to the center of degenerated caudal discs 2 weeks after modeling. The amount of viable MSCs remained in the disc was quantified by measuring GFP intensity using in vivo fluorescence (Fig. 5A). Signal intensities in discs injected with LbL-MSCs were significantly higher than those injected with MSCs at 1, 3, 7, and 14 d after cell administration (Fig. 5B and D), although the fluorescence signal can barely be detected at d14. Immunofluorescence staining against GFP further confirmed longer retention of LbL-MSCs than MSCs in degenerated discs. At d1, similar ratios of GFP-labeled cells were observed in MSC- and LbL-MSC-treated discs. However, more GFP-labeled cells were identified in LbL-MSC-treated discs at 3, 7, and 14 d after injection, as compared with those treated with MSCs (*P* < 0.001, *P* < 0.05, and *P* < 0.001, respectively) (Fig. 5C and E). Results suggested that cell surface microgel coating prolonged MSC retention in degenerated discs*.*

### Microgel coating improved reparative potency of MSCs in treating disc degeneration

**Fig. 6.**
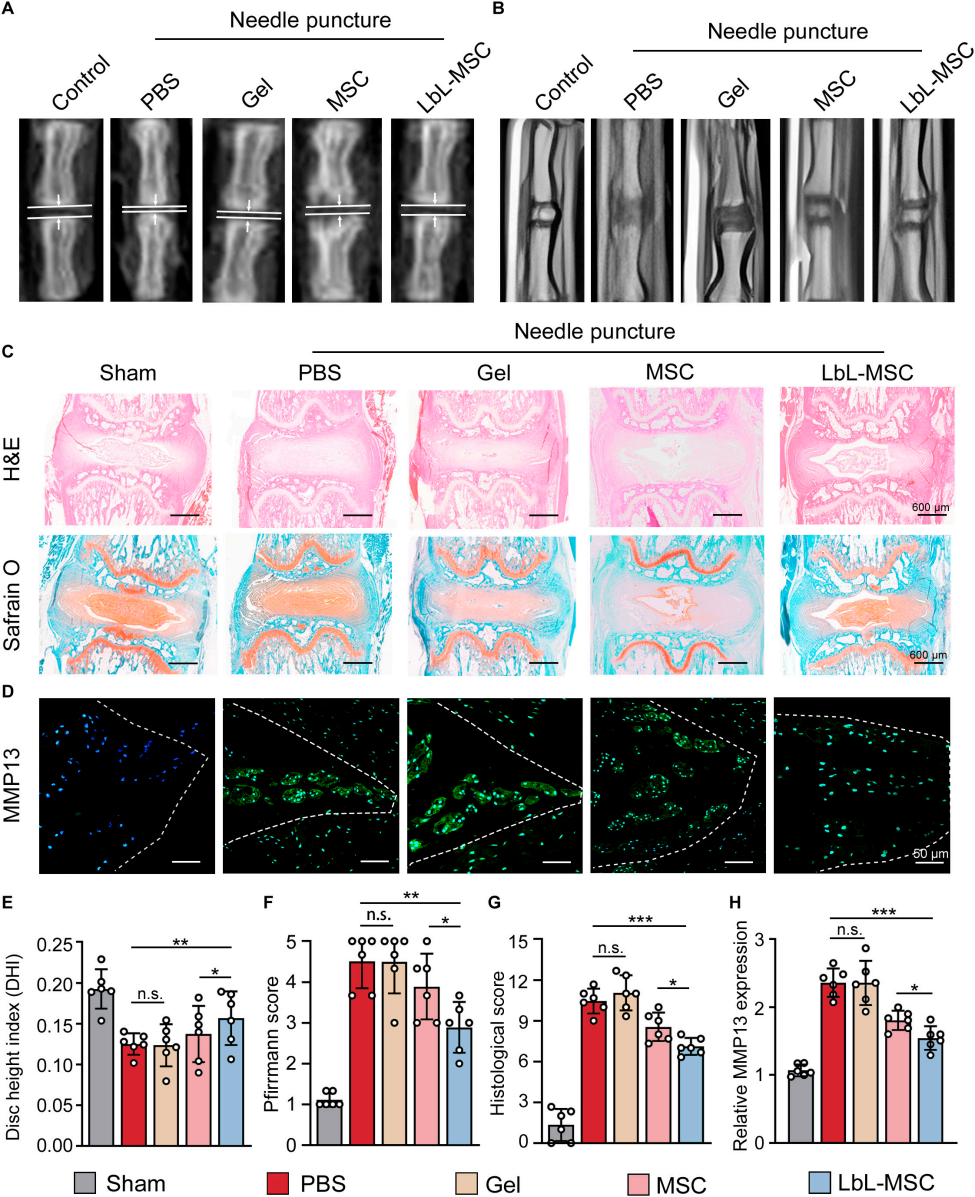
Microgel-encapsulated MSCs attenuated needle-puncture-induced disc degeneration in rats. (A) Representative x-ray and (B) MR images from the 6 experimental groups 8 weeks after needle puncture. (C) HE, SO staining (scale bars, 600 μm), and (D) immunofluorescence staining of MMP13 in experimental discs (scale bars, 50 μm). (E) DHI measured on x-ray images. (F) Pfirrmann score assessed on MR images. (G) Histology score measured on HE staining. (H) Relative MMP13 expression: Immunofluorescence results in different treatment groups. *N* = 6 for each group. *n* = 5 independent experiments. *P < 0.05; **P < 0.01; ***P < 0.001.

As microgel coating on MSCs improved cell survival and retention in degenerated discs, we further examine whether encapsulation can enhance the reparative potency. Intradiscal injection of phosphate-buffered saline (PBS), hydrogel (alginate and gelatin), MSC, and LbL-MSC was performed in the rat model of disc degeneration. X-ray and magnetic resonance imaging (MRI) were used to assess disc degeneration 8 weeks after treatment (Fig. 6A and B). The modeled discs treated with MSCs and LbL-MSCs had significantly greater disc height index (DHI) measurements than the PBS and hydrogel groups, and LbL-MSC-treated discs had higher DHI than MSC-treated discs (Fig. 6E). Both MSC- and LbL-MSC-treated discs exhibited greater signal intensity on T2-weighted MR images than the PBS- and hydrogel-treated discs. Moreover, discs treated with MSCs had less Pfirrmann score than PBS- and hydrogel-treated discs (*P* < 0.05 for both; Fig. 6F). In addition, the LbL-MSC-treated discs had less Pfirrmann score than MSC-treated discs (*P* < 0.05), suggesting that LbL-MSCs performed better than MSCs in alleviating disc degeneration.

Hematoxylin–eosin (HE) and Safranin O/fast green (SO) staining were used to evaluate the morphological change of the intervertebral disc (Fig. 6C and G). On HE staining, discs in sham group were with full NP tissues, and annulus fibrosus remained intact. Needle puncture altered disc structure seriously. In PBS and hydrogel groups, the gel-like nucleus was replaced by disorganized fibrocartilaginous tissues, and the annulus became obscure, with concomitant disc narrowing. MSC transplantation partially sustained the gel-like NP, suggesting that MSCs alleviated disc degeneration. LbL-MSC showed an even better result than MSC treatment, with more normal nucleus and intact surrounding annulus. SO staining revealed that discs treated with PBS or hydrogel exhibited a reduced proteoglycan-rich matrix and a loss of collagen contents. LbL-MSC-treated discs exhibited relatively intense staining than other groups, indicating that microgel-encapsulated MSCs better maintained proteoglycan and collagen contents.

Histological score, which takes structure integrity and tissue components into account, was measured. PBS and hydrogel treatments had no therapeutic effect on degenerated discs, while MSC treatment substantially ameliorated morphological degeneration, and LbL-MSC treatment fared better than bare MSC treatment. Further, immunofluorescence showed decreased levels of matrix metalloproteinase 13 (MMP13) in MSC- and LbL-MSC-treated discs, as compared to PBS and hydrogel treatments (*P* < 0.05), with the LbL-MSC-treated discs having the lowest expression level (*P* < 0.001) (Fig. 6D and H). Findings suggested that microgel encapsulation was able to improve the therapeutic effects of MSCs in treating disc degeneration.

### MSCs attenuated tissue pyroptosis, and microgel improved therapeutic effect of MSC treatment

**Fig. 7.**
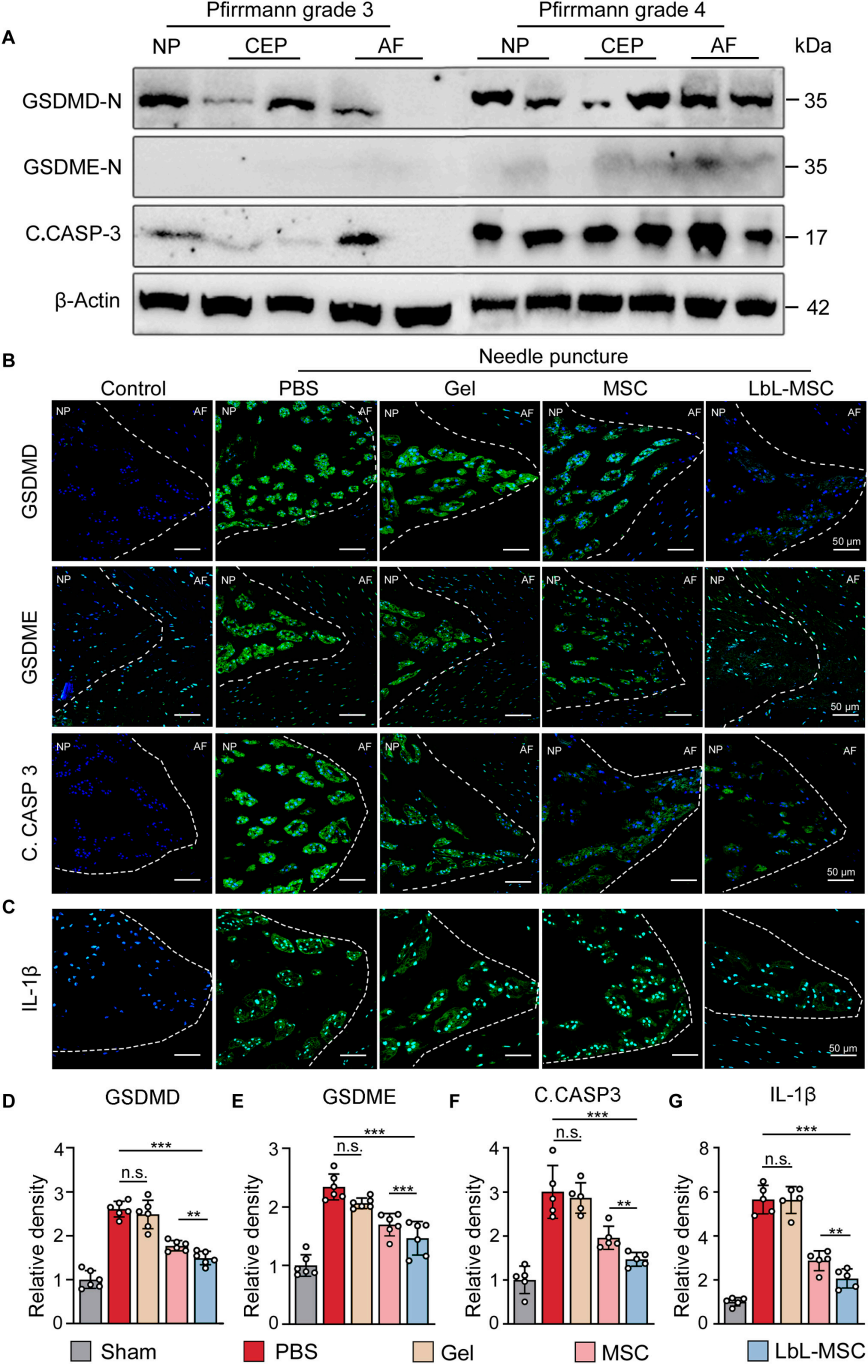
LbL-MSCs suppressed pyroptosis in disc degeneration. (A) The expression of pyroptosis-related proteins in human lumbar disc tissues with Pfirrmann grades 3 and 4. NP, nucleus pulposus; CEP, cartilage endplate; AF, annulus fibrosus. (B) Immunofluorescence analysis of pyroptosis-related proteins. (C) IL-1β in a rat model of needle-puncture-induced disc degeneration. Scale bars, 50 μm. (D to G) Quantitative analyses of protein expression. *n* = 5 independent experiments. **P < 0.01; ***P < 0.001.

As previous studies suggested that pyroptosis was associated with the development of degenerative disease, we obtained disc tissues from 6 surgical patients to examine the relationship between pyroptosis and disc degeneration. Western blotting showed that discs with Pfirrmann grade 4 exhibited higher expression of pyroptosis-related proteins than the discs with grade 3 (Fig. 7A).

Using the rat model, we hence investigated whether MSC therapy can attenuate pyroptosis in disc degeneration. Needle-puncture-induced model of disc degeneration was established, and various treatments were applied. Disc samples were analyzed 8 weeks after modeling. Immunofluorescence analysis revealed pyroptosis activation in the NP, indicating that pyroptosis may play a role in disc degeneration. Compared to the PBS and hydrogel treatments, MSC treatment alleviated pyroptosis activation (Fig. 7B and D to F) and IL-1β release in the modeled discs, indicating a suppressed inflammation there (Fig. 7C and G). In addition, LbL-MSC treatment demonstrated a stronger inhibition of pyroptosis and IL-1β release than MSC did. Collectively, findings suggested that microgel-encapsulated MSC attenuated pyroptosis in the progression of disc degeneration (Fig. 1C).

## Discussion

With an LbL self-assembling scaffold technique, microgel was developed to encapsulate MSCs and used as seed cells to treat disc degeneration. Microgel-coated MSCs exhibited enhanced cell viability, sustained cellular functions, and increased survive rate in vivo. Under oxidative stress, microgel can reduce the death of transplanted MSCs in the disc via suppressing pyroptosis and maintaining mitochondria homeostasis and, thus, effectively treat disc degeneration. Findings provided a new strategy to improve therapeutic efficacy of cell therapy in treating disc degeneration.

Microgel, which can reduce cell damage resulting from mechanical force [48], may help coated cells to resist the shear stress in the disc. Moreover, microgel can also serve as a scaffold for MSC therapy and reduce physical stimulation from the degenerated ECM. While alterations in membrane curvature and tension can interrupt the formation and function of cell membrane pores related to neutrophil extracellular traps [58], it is likely that microgel can modulate cell membrane curvature to regulate the opening of pyroptosis pores and extracellular osmotic pressure, resulting in decreased cytokines flow through the membrane pores.

Different from other tissues, the disc is sensitive to mechanical loading. While high mechanical stress promotes NP fibrosis and ECM loss [59,60], intradiscal application of the traditional bulk hydrogel might exert aberrant mechanic stress to adjacent disc tissues, which may lead to exaggerated tissue degeneration [60,61]. The complicated tissue architecture of the disc also made it difficult for bulk hydrogel to exhibit real-time mechanical functions as needed [45,46]. Hence, the mismatched hydrogel mechanical properties may impair the physiological status of transplanted cells and limit therapeutic efficacy [62,63]. Different from the bulk hydrogel, microgel proposed in our study was in small size, which allowed encapsulated cells to perceive mechanical properties of the surrounding tissue while exerting cytoprotective effects [47]. As mechanical stimulation from local microenvironment regulated MSC activity and promoted tissue regeneration [64,65], microgel might be more compatible than bulk hydrogel in the disc repairing.

On the other hand, MSC migration and homing, important steps for cell therapy and tissue regeneration, are under accurate regulation [42,43]. Although not fully understood, the tight cross-linked structure of the conventional bulk hydrogels and the narrow pore size might hinder the homing of MSCs [40,66]. As single-cell hydrogel encapsulation sustained the independence of single cell and minimized the hydrogel hindrance [47], microgel retained MSC homing and migration ability and allowed transplanted MSCs to accommodate to the disc. Microgel-coated MSCs migrated in respond to chemokines and cellular factors from endogenous niche rather than within the hydrogel [67]. MSC niche regulated cellular function with respect to gene expression and differentiation capacities, resulting in effective and long-lasting regeneration [68,69]. Besides, the simple encapsulation method and materials made clinical translation feasible and inexpensive. Hence, microgel may be a promising way to fully exhibit the therapeutic effect of MSC therapy.

Transcriptome profiling demonstrated that in addition to pyroptosis, other cell death pathways might also participate in cytoprotective ability of microgel coating. The concept of “PANoptosis” has been proposed to explain the communications between various pathways of cell death. Elements of different cell death formed a putative PANoptosome, which acted as a central cell death complex to initiate GSDM-mediated pyroptosis-like death [70]. Our results revealed that oxidative stress induced pyroptosis in MSCs with coactivation of multiple cell death pathways, indicating potential PANoptosis involvement. Microgel attenuated pyroptosis activation and suppressed multiple cell death pathways. Therefore, we hypothesized that microgel might suppress PANoptosis to improve cell survival. However, the lack of mechanism study and reliable method to detect PANoptosis limit in-depth study. PANoptosis thus may be the target for improving the efficacy of cell therapy, although further study is needed.

Last, pyroptosis activation was detected in patients with severe disc degeneration, indicating possible involvement of pyroptosis in pathological transformation. Although comprehensive illustration of pyroptosis to disc degeneration has not been fully demonstrated, it is generally believed that abnormal pyroptosis activation exaggerates disc degeneration [71] through overexpression of inflammatory factors and MMP [72,73]. Therefore, pyroptosis inhibition might be a potential strategy to disc degeneration therapy. Drug treatment is currently the most dominant approach for pyroptosis inhibition, however, exhibited high incidence of side effects [74]. Our study revealed that MSC treatment attenuated pyroptosis and ameliorated the condition of oligocytes and ECM loss in degenerated discs, suggesting that cell therapy might be a better solution for pyroptosis suppression in discs. Microgel coating improved the therapeutic efficacy with enhanced pyroptosis suppression ability of LbL-MSCs. Further studies are needed to elucidate the therapeutic effects and mechanism of pyroptosis inhibition to disc degeneration.

There are still some limitations that should be considered. In addition to cell delivery, microgel also acts as a scaffold for structural support. The effects and mechanism of microgel to regulate cell secretion, growth, and differentiation deserve further investigation. Charge interaction between hydrogel components could potentially be interrupted by disc microenvironment. The electrical properties of components could be changed in degenerated discs with decreased pH and oxidative stress, thus might be reducing the strength of charge interaction and disabling the protection of microgel. The stability of microgel in an acidic and oxidative degeneration environment is an issue that needs to be discussed. Besides, it was hard to track the transplanted cell in vivo for a long period of time, especially in discs. Monitoring the hydrogel coating of cells was even harder because of the small size of hydrogel layer. Therefore, the fate of microgel-coated MSCs in degenerated discs was difficult to investigate*.* Advanced analysis approaches might help to illustrate the impact of microgel on transplanted MSCs in vivo during practical application. These limitations might be overcome through seeking technical cooperation and improvements in the future.

## Conclusion

In this study, we demonstrated a biocompatible microgel to improve the cell therapy efficacy in degenerated disc treatment for the first time. LbL fabrication method was used to construct microgel coating on the surface of individual MSC. With enhanced cell survival, microgel-coated MSCs showed prolonged in vivo retention and improved effect on alleviating disc degeneration. Our study provided a new perspective to the improvement of cell therapy efficacy and shed light on the development of disc degeneration treatment strategies.

## Materials and Methods

### Study design

The objectives of this study were to investigate the potent of microgel in enhancing MSC therapeutic effects in disc degeneration treatment and its possible protective mechanism. Here, we first encapsulated MSCs through LbL fabrication method (LbL-MSCs) and examined MSC survival in oxidative stress for mimicking microenvironment of degenerated intervertebral discs. It was revealed that microgel coating improved cell survival without altering cellular function. The function of microgel in the inhibition of pyroptosis activation and sustainment of mitochondria homeostasis was comprehensively studied in vitro to investigate cytoprotective mechanism of microgel. Then, we analyzed the function of microgel coating to MSC therapy in treating disc degeneration in the needle puncture rat model. MSC retention in degenerated discs was detected through fluorescence signal intensity of cells. Therapeutic effects of MSCs to disc degeneration was evaluated by radiology and histological analysis. For each experiment, sample sizes for independent experiments are indicated in the figure legend. All studies involving human samples and animals were approved by ethical review board at the author’s institute. Written consents were obtained from all patients before operation.

### Chemicals

Alginate and gelatin were obtained from Aladdin Biochemical Reagent. *Tert*-butyl hydroperoxide and CCCP were purchased from Sigma-Aldrich. All other chemicals were of analytical grade and used as received, if not otherwise described. Water was purified by the Milli-Q system in the experiments.

### Assessment of human intervertebral disc samples

All experiments related to human samples were approved by the ethical review board at the author’s institute (ethical approval number ZDYY-2020-333). Each patient understood the study protocol and signed a written consent. Intervertebral disc specimens were obtained from 4 patients who underwent lumbar disc discectomy surgery. Disc tissues were used for Western blotting to study the activation of pyroptosis in disc degeneration.

### Animals

Sprague–Dawley rats were purchased from the Animal Center at the author’s institute. In the animal facility at the author’s institute, rats were bred, housed, and used under a specific-pathogen-free environment. All animal studies were performed with the approval of the ethical review board at the author’s institute (ethical approval number ZDYY-2022-53).

### MSC isolation and culture conditions

The tibias of 3-week-old Sprague–Dawley rats (Zhejiang Academy of Medical Sciences Animal Center, China) were flushed with PBS to collect the bone marrow cells. Then, the cell mixture was centrifuged, and the supernatant was discarded. The collected cells were resuspended and cultured in MSC culture medium consisting of α-minimum essential medium (90%, v/v; Meilunbio, MA0216), fetal bovine serum (10%, v/v; Beyotime, C0232), and penicillin–streptomycin (1%, v/v; Beyotime, C0222). The medium was half changed 2 d later and was completely changed for another 2 d later to let MSCs fully adhered to the disc and remove the non-MSC population. MSCs were characterized by tri-series differentiation. Cells were cultivated under standard culture conditions in α-minimum essential medium and 10% fetal bovine serum under 21% oxygen and 5% carbon dioxide. Pyroptosis was induced by adding 300 μM TBHP and incubating cells for 4 h.

### LbL single-cell encapsulation

Cultivated MSCs were centrifuged to remove the medium within a 15-ml centrifuge tube. For 2 × 10^6^ MSCs, 1 ml of 0.1% gelatin solution was added, and the tube was gently shaken for 10 min. Then, the tube was centrifuged at 1,000 rpm for 5 min, and the supernatant was discarded. Cells were washed by PBS once, and the tube was centrifuged again to remove the PBS. After that, cells were incubated with 1 ml of 0.1% alginate for 10 min and centrifuged again to collect cells. The process was repeated to coat another layer of gelatin to form a 3-layer LbL encapsulation of MSCs.

For hydrogel labeling with fluorescence, 20 mg of gelatin (Aladdin, G108396) was dissolved in 2 ml of 0.1 M sodium bicarbonate buffer, and 10 mg of FITC (Aladdin, F272901) was dissolved in 1 ml of dimethyl sulfoxide (Aladdin, F272901). FITC solution was gently added into the magnetically stirring gelatin solution. The reaction was incubated overnight at room temperature with continuous stirring to prepare the gelatin–FITC. Then, the solution was dialyzed for 3 d. Ten milligrams of alginate (Aladdin, S278630) was dissolved in 1 ml of PBS and activated by 10 mg of 1-ethyl-3-(3-dimethylaminopropyl)carbodiimide (Aladdin, E106172) for 30 min. Then, 3 mg of ethanediamine (Aladdin, E300209) was added. The mixture was stirred overnight at room temperature. The solution was firstly dialyzed for 1 d, and alginate–ethanediamine solution was obtained. Then, the powder was put into 2 ml of RhoB (Aladdin, R104961) solution (2.5 mg/ml in PBS). The mixture was stirred overnight at room temperature. The alginate–rhodamine was obtained after dialyzed for 3 d.

The hydrogel encapsulation over cell surface was directly characterized by TEM and SEM. Fluorescence analysis by microscope and flow cytometry was conducted for encapsulation efficiency.

### Size and aggregation analysis

After encapsulated with FITC-labeled gelatin, LbL-MSCs were rinsed with PBS for 2 times and centrifuged to remove excess gelatin solution. The size of nanogel-coated MSCs is measured by Mastersizer 3000 laser particle size analyzer (Malvern Instruments Ltd.). Cell aggregation was evaluated by flow cytometry and fluorescence intensities at wavelength of 488 nm were analyzed to evaluate the encapsulation efficiency.

### Cell survival

Cell survival was detected with calcein-acetoxymethyl ester (AM)/propidium iodide (PI) staining (Keygene, KGAF001), and the fluorescence was read using a microplate reader. Briefly, MSCs and LbL-MSCs were seeded in 24-well plates and treated with 300 μg of TBHP for 4 h. Medium was replaced by calcein-AM/PI working mixture and cultivated in 37 °C for another 30 min. Fluorescence images were captured with a fluorescence inverted microscope (Olympus, IX83-FV3000). Death rate was calculated as the number of cells labeled with PI, divided by the total number of cells identified with calcein-AM and PI.

### RNA sequencing

Total RNA was isolated from 3 replicates of MSC or LbL-MSC group with a RNeasy mini kit (QIAGEN) and sent to Huada (Wuhan, China) for preparation, sequencing, and mapping. The data were evaluated via hierarchical clustering analysis, pathway analysis, and cluster analysis.

### Western blotting

Cells were rinsed with cold PBS, lysed in 5× SDS gel loading buffer, and boiled for 10 min; then, the proteins in the lysate were separated on 12% SDS polyacrylamide gels, electrotransferred to polyvinylidene difluoride membranes (Millipore, Boston), and stained with the following primary antibodies: GSDMD (1:1,000; Abcam, ab219800), GSDME (1:1,000; Abcam, ab215191), cleaved caspase-3 (1:1,000; Cell Signaling Technology, #9664), and β-actin (1:1,000; Abcam, ab8226). Primary antibodies were stained with horseradish-peroxidase-conjugated secondary antibodies (1:10,000; Abcam, ab205718) and visualized with a chemiluminescence ECL Western blotting system (Millipore)

### Immunofluorescence staining

Fixed cells or sections were washed with PBS, permeabilized with 0.2% Triton X-100 (Sigma-Aldrich) for 20 min, and blocked with 5% bovine serum albumin for 1 h at 37 °C. Cells or sections were then incubated overnight at 4 °C with primary antibodies: GSDMD (1:1,000; Abcam, ab219800), GSDME (1:1,000; Abcam, ab215191), cleaved caspase-3 (1:1,000; Cell Signaling Technology, #9664), GFP (1:50; Beyotime, AF0159), and MMP13 (1:100; Abcam, 39012), followed by incubation with secondary antibodies conjugated to Alexa Fluor 488 or Alexa Fluor 555 (1:200) for 1 h at room temperature. Nuclei were counterstained with 4′,6-diamidino-2-phenylindole (DAPI; Beyotime, C1006) for 15 min. Staining results were examined under a fluorescence microscope (Olympus, IX83-FV3000) and quantified using ImageJ (Bethesda, MD, V1.8.0, National Institutes of Health).

### LDH measurement

LDH release was determined as an indicative of pyroptosis using the LDH Cytotoxicity Assay Kit (Beyotime, C0016). TBHP (300 μM) was used to treat MSCs, Mix-MSCs, and LbL-MSCs for 4 h, and cell culture supernatant was collected. LDH release was analyzed according to the manufacturer’s protocol and normalized to the released LDH of untreated MSCs.

### Assessment of mitochondrial membrane potential

JC-1 staining kit (Beyotime, C2003S) was used to assess the mitochondrial membrane potential. Briefly, MSCs were seeded in a 24-well plate with a density of 5 × 10^4^ cells per well and cultured for 24 h. After TBHP stimulation for 4 h, the medium was replaced with fresh medium and then stained with JC-1 according to the manufacturer’s instructions. The cells were washed twice with JC-1 washing liquid. Fluorescence of mitochondria was imaged by confocal fluorescence microscope (Olympus, IX83-FV3000) and analyzed by flow cytometry (Beckman Coulter).

### mtDNA release analysis

MitoTracker (Beyotime, C1035) was used to label cell mitochondria, and dsDNA was labeled by immunofluorescence. Briefly, MSCs were seeded in a 24-well plate with a density of 5 × 10^4^ cells per well and cultured for 24 h. After TBHP stimulation for 2 h, the medium was replaced with fresh medium. Normal MSCs or treated MSCs were first stained with MitoTracker staining. The cells were then washed twice with PBS. After fixation and blockage, the cells were incubated overnight at 4 °C with anti-dsDNA (1:250; Abcam, ab273137). Subsequently, a fluorescence-labeled secondary antibody was added and incubated with the cells for 1 h, and the nuclei were stained with DAPI. The colocalization of MitoTracker and dsDNA fluorescence signals was analyzed by ImageJ.

### Mitochondria dysfunction and cyclosporin A treatment

Mitochondrial depolarization was induced by treating MSCs and LbL-MSCs with 20 μM CCCP for 2 h. Cyclosporin A (1 μM) was pretreated before CCCP treatment to alleviate the dysfunction of mitochondria. Western blotting and immunofluorescence were then conducted to analyze the pyroptosis-related protein activation.

### Cytosolic mtDNA copy number analysis

Mitochondria were isolated from MSCs, Mix-MSCs, and LbL-MSCs using a mitochondrial isolation kit (Beyotime, C3601). After the mitochondria were discarded, the cytoplasm was prepared to isolate cytosolic mtDNA using a genomic DNA miniprep kit (Tiangen, DP304). Polymerase chain reaction quantification was then carried out to analyze mtDNA and nuclear DNA in the cytoplasm. mtDNA was marked by tRNA-LeuUUR, while nuclear DNA was characterized by β-microglobulin genes. The ratio of mtDNA to nuclear DNA in the cytoplasm represented the copy number of cytosolic mtDNA.

### Needle-puncture-induced disc degeneration in rats

Thirty female Sprague–Dawley rats (3 months old, approximately 150 g) purchased from the Institutional Animal Center were used to establish a disc degeneration model and appropriate controls. Briefly, rats were anesthetized intraperitoneally with 0.8% (w/v) pentobarbital sodium (10 μl/g of body weight). The intervertebral discs between coccyx vertebra 5 and 6, 6 and 7, 7 and 8 (Co5/6, Co6/7, and Co7/8) were identified and marked on the skin for puncture. For model establish, discs were percutaneously punctured using a 29-gauge needle through the skin mark to a depth of about 2 mm. The needle was then rotated 360° and maintained inside the disc for 1 min before removal.

Rats were randomly divided into control (no disc puncture, *n* = 6) and experiment groups (disc puncture, *n* = 24). Rats in both groups were fed regular food and water at room temperature for 8 weeks. Experiment groups were divided into 4 groups, and each were intradiscally injected with PBS, hydrogel components, MSCs, or LbL-MSCs respectively, into the Co5/6, Co6/7, and Co 7/8. Thereafter, animals were sacrificed, and disc tissues were collected.

### GFP-labeled MSC construction

Cultivated MSCs of P2 (1 × 10^6^) were infected with GFP-encoding recombinant lentiviruses (Genechem Company) in polybrene (10 μg/ml; Beyotime, C0351) for 12 h; then, the growth medium was replaced. The cells were cultivated for 48 h after virus transfection, and the culture medium was replaced by medium with puromycin (5 μg/ml; Beyotime, ST551). Cells were examined daily and observed for the percentage of live cell growth, as well as the level and percentage of GFP expression. MSCs were collected for in vivo tracking when all surviving cells stably express GFP fluorescent signal. The optimal duration of puromycin screen is between 3 and 10 d.

### Transplanted cell retention

Thirty rats were randomly divided into MSC group (*n* = 15) and LbL-MSC groups (*n* = 15). All the rats were modeled with needle-puncture-induced intervertebral disc degeneration in Co5/6 and Co6/7 discs. GFP-labeled MSCs or LbL-MSCs were then injected into the discs. Zero, 1, 3, 7, and 14 d after injection, 3 rats from each of the 2 groups were sacrificed and discs (*n* = 6 each group) were collected. In vivo fluorescence images were acquired with an in vivo imaging system (PerkinElmer), and immunofluorescence was conducted to analyze the GFP-MSC signal.

### Radiographic evaluation

X-ray and MRI were conducted 8 weeks after modeling. X-ray was taken for rat tails, and radiographs were measured to acquire disc height using ImageJ. The disc height was expressed as the DHI based on the method of Masuda et al. [75]. The average DHI was calculated by averaging the measured disc heights obtained from the anterior, middle, and posterior portions of the intervertebral disc degeneration and divided by the average of adjacent vertebral body heights. MRI was taken using a 3.0-T Intera Achieva 3.0 MR scanner (Philips) to acquire T2-weighted MR images. Imaging protocol is as follows: spin echo repetition time, 2,700 ms; echo time, 99 ms; number of excitations, 8; field of view, 5 cm; slice thickness, 1.5 mm. Three surgeons who were blinded to animal treatment history assessed degree of disc degeneration using the Pfirrmann scale [76], which is based on structure, signal intensity distribution, and height of the intervertebral disc (with grade I indicating least degeneration and grade V most severe degeneration)

### Histological evaluation of disc degeneration

Harvested rat discs were fixed in 4% buffered paraformaldehyde for 24 h, decalcified in 10% EDTA (Solarbio, E8030) solution for 1 month, embedded in paraffin, and cut into 5-μm sections. SO staining and HE staining were performed to evaluate the degree of disc degeneration. Images of stained sections were evaluated by 3 independent raters who are blinded to the research protocol, and measurements were averaged for statistical analysis. Histological score was rated on the basis of cellularity, morphology, structure, and border of the annulus fibrosus and NP regions [77], with a score of 0 to 3 indicating normal disc, 4 to 10 as moderately degenerated disc, and 11 to 16 as severely degenerated disc.

### Statistical analysis

Unpaired one-tailed *t* test was used to determine the difference between 2 groups, and one-way analysis of variance (ANOVA) and Tukey correction for comparisons were used when there are 3 groups or more. Normality of data distribution was assessed using the Shapiro–Wilk test. Statistical analyses were performed using GraphPad Prism (GraphPad Software, version 8.0.0, San Diego, USA).

## Data Availability

The data that support the findings of this study are available on request from the corresponding author upon reasonable request.

## References

[1] Battié MC, Ortega-Alonso A, Niemelainen R, Gill K, Levalahti E, Videman T, Kaprio J. Brief report: Lumbar spinal stenosis is a highly genetic condition partly mediated by disc degeneration: Lumbar spinal stenosis is highly genetic. Arthritis Rheumatol. 2014;66:3505–3510. 25155712 10.1002/art.38823 PMC4308556

[2] Krock E, Millecamps M, Anderson KM, Srivastava A, Reihsen TE, Hari P, Sun YR, Jang SH, Wilcox GL, Belani KG, et al. Interleukin-8 as a therapeutic target for chronic low back pain: Upregulation in human cerebrospinal fluid and pre-clinical validation with chronic reparixin in the SPARC-null mouse model. EBioMedicine. 2019;43:487–500. 31047862 10.1016/j.ebiom.2019.04.032 PMC6558025

[3] Adams MA, Roughley PJ. What is intervertebral disc degeneration, and what causes it? Spine. 2006;31(18):2151–2161. 16915105 10.1097/01.brs.0000231761.73859.2c

[4] Feng C, Yang M, Lan M, Liu C, Zhang Y, Huang B, Liu H, Zhou Y. ROS: Crucial intermediators in the pathogenesis of intervertebral disc degeneration. Oxidative Med Cell Longev. 2017;2017:5601593. 10.1155/2017/5601593 PMC5368368 28392887

[5] Wu PH, Kim HS, Jang I-T. Intervertebral disc diseases PART 2: A review of the current diagnostic and treatment strategies for intervertebral disc disease. Int J Mol Sci. 2020;21(6):2135. 32244936 10.3390/ijms21062135 PMC7139690

[6] Zhu B, Shang L, Han X, Li X, Wang H, Sang P, Lv C, Li J, Liu X. Revision surgery for symptomatic postoperative pseudocyst following full-endoscopic lumbar discectomy: Clinical characteristics and surgical strategies. BMC Musculoskelet Disord. 2022;23:835. 36057592 10.1186/s12891-022-05791-y PMC9440536

[7] Sun S, Wang L, Xue Y. “Inside disc out” discectomy for the treatment of discogenic lumbar spinal canal stenosis under the intervertebral foramen endoscope. Orthop Surg. 2022;15(1):355–361. 36398485 10.1111/os.13550 PMC9837259

[8] Lee CK, Heo DH, Chung H, Roh EJ, Darai A, Kyung JW, Choi H, Kwon SY, Bhujel B, Han I. Advances in tissue engineering for disc repair. Appl Sci. 2021;11(4):1919.

[9] Wu D, Li G, Zhou X, Zhang W, Liang H, Luo R, Wang K, Feng X, Song Y, Yang C. Repair strategies and bioactive functional materials for intervertebral disc. Adv Funct Mater. 2022;32(52):2209471.

[10] Dörnen J, Dittmar T. The role of MSCs and cell fusion in tissue regeneration. Int J Mol Sci. 2021;22:10980. 34681639 10.3390/ijms222010980 PMC8535885

[11] Krut Z, Pelled G, Gazit D, Gazit Z. Stem cells and exosomes: New therapies for intervertebral disc degeneration. Cell. 2021;10(9):2241. 10.3390/cells10092241 PMC8471333 34571890

[12] Urits I, Capuco A, Sharma M, Kaye AD, Viswanath O, Cornett EM, Orhurhu V. Stem cell therapies for treatment of discogenic low back pain: A comprehensive review. Curr Pain Headache Rep. 2019;23(9):65. 31359164 10.1007/s11916-019-0804-y

[13] Orozco L, Soler R, Morera C, Alberca M, Sánchez A, García-Sancho J. Intervertebral disc repair by autologous mesenchymal bone marrow cells: A pilot study. Transplantation. 2011;92(7):822–828. 21792091 10.1097/TP.0b013e3182298a15

[14] Ossendorff R, Walter SG, Schildberg FA, Khoury M, Salzmann GM. Controversies in regenerative medicine: Should knee joint osteoarthritis be treated with mesenchymal stromal cells? Eur Cell Mater. 2022;43:98–111. 35298024 10.22203/eCM.v043a09

[15] Kobayashi H, Tohyama S, Kanazawa H, Ichimura H, Chino S, Tanaka Y, Suzuki Y, Zhao J, Shiba N, Kadota S, et al. Intracoronary transplantation of pluripotent stem cell-derived cardiomyocytes: Inefficient procedure for cardiac regeneration. J Mol Cell Cardiol. 2023;171:77–87. 10.1016/j.yjmcc.2022.11.004 36403760

[16] Urban JPG. The role of the physicochemical environment in determining disc cell behaviour. Biochem Soc Trans. 2002;30(Pt 6):858–863. 12440933 10.1042/bst0300858

[17] Maidhof R, Rafiuddin A, Chowdhury F, Jacobsen T, Chahine NO. Timing of mesenchymal stem cell delivery impacts the fate and therapeutic potential in intervertebral disc repair. J Orthop Res. 2017;35(1):32–40. 27334230 10.1002/jor.23350

[18] Vadalà G, Sowa G, Hubert M, Gilbertson LG, Denaro V, Kang JD. Mesenchymal stem cells injection in degenerated intervertebral disc: Cell leakage may induce osteophyte formation. J Tissue Eng Regen Med. 2012;6(5):348–355. 21671407 10.1002/term.433

[19] Varden LJ, Nguyen DT, Michalek AJ. Slow depressurization following intradiscal injection leads to injectate leakage in a large animal model. JOR Spine. 2019;2(3): e1061. 31572978 10.1002/jsp2.1061 PMC6764785

[20] Daly C, Ghosh P, Jenkin G, Oehme D, Goldschlager T. A review of animal models of intervertebral disc degeneration: Pathophysiology, regeneration, and translation to the clinic. BioMed Res Int. 2016;2016:5952165. 27314030 10.1155/2016/5952165 PMC4893450

[21] Sakai D, Grad S. Advancing the cellular and molecular therapy for intervertebral disc disease. Adv Drug Deliv Rev. 2015;84:159–171. 24993611 10.1016/j.addr.2014.06.009

[22] Nicholson DW. From bench to clinic with apoptosis-based therapeutic agents. Nature. 2000;407(6805):810–816. 11048733 10.1038/35037747

[23] Bredesen DE, Rao RV, Mehlen P. Cell death in the nervous system. Nature. 2006;443(7133):796–802. 17051206 10.1038/nature05293 PMC3970704

[24] Pasparakis M, Vandenabeele P. Necroptosis and its role in inflammation. Nature. 2015;517(7534):311–320. 25592536 10.1038/nature14191

[25] Oberst A, Dillon CP, Weinlich R, McCormick LL, Fitzgerald P, Pop C, Hakem R, Salvesen GS, Green DR. Catalytic activity of the caspase-8–FLIPL complex inhibits RIPK3-dependent necrosis. Nature. 2011;471(7338):363–367. 21368763 10.1038/nature09852 PMC3077893

[26] Li Z, Chen S, Ma K, Lv X, Lin H, Hu B, He R, Shao Z. CsA attenuates compression-induced nucleus pulposus mesenchymal stem cells apoptosis via alleviating mitochondrial dysfunction and oxidative stress. Life Sci. 2018;205:26–37. 29746847 10.1016/j.lfs.2018.05.014

[27] Sakai D, Andersson GBJ. Stem cell therapy for intervertebral disc regeneration: Obstacles and solutions. Nat Rev Rheumatol. 2015;11(4):243–256. 25708497 10.1038/nrrheum.2015.13

[28] Ding J, Wang K, Liu W, She Y, Sun Q, Shi J, Sun H, Wang DC, Shao F. Pore-forming activity and structural autoinhibition of the gasdermin family. Nature. 2016;535(7610):111–116. 27281216 10.1038/nature18590

[29] Feng Y, Li M, Yangzhong X, Zhang X, Zu A, Hou Y, Li L, Sun S. Pyroptosis in inflammation-related respiratory disease. J Physiol Biochem. 2022;78(4):721–737. 35819638 10.1007/s13105-022-00909-1 PMC9684248

[30] Sordi MB, Magini de SR, Panahipour L, Gruber R. Pyroptosis-mediated periodontal disease. Int J Mol Sci. 2021;23(1):372. 35008798 10.3390/ijms23010372 PMC8745163

[31] Tang D, Kang R, Berghe TV, Vandenabeele P, Kroemer G. The molecular machinery of regulated cell death. Cell Res. 2019;29(5):347–364. 30948788 10.1038/s41422-019-0164-5 PMC6796845

[32] Mata R, Yao Y, Cao W, Ding J, Zhou T, Zhai Z, Gao C. The dynamic inflammatory tissue microenvironment: Signality and disease therapy by biomaterials. Research. 2021;2021: 4189516. 33623917 10.34133/2021/4189516 PMC7879376

[33] Li X, Zhang P, Yin Z, Xu F, Yang Z-H, Jin J, Qu J, Liu Z, Qi H, Yao C, et al. Caspase-1 and Gasdermin D afford the optimal targets with distinct switching strategies in NLRP1b inflammasome-induced cell death. Research. 2022;2022: 9838341. 35958114 10.34133/2022/9838341 PMC9343085

[34] Yadav SK, Kambis TN, Kar S, Park SY, Mishra PK. MMP9 mediates acute hyperglycemia-induced human cardiac stem cell death by upregulating apoptosis and pyroptosis in vitro. Cell Death Dis. 2020;11(3):186. 32170070 10.1038/s41419-020-2367-6 PMC7070071

[35] Lee CY, Lee S, Jeong S, Lee J, Seo H-H, Shin S, Park JH, Song BW, Kim IK, Choi JW, et al. Suppressing pyroptosis augments post-transplant survival of stem cells and cardiac function following ischemic injury. Int J Mol Sci. 2021;22(10):7946. 34360711 10.3390/ijms22157946 PMC8348609

[36] Zhou T, Yuan Z, Weng J, Pei D, Du X, He C, Lai P. Challenges and advances in clinical applications of mesenchymal stromal cells. J Hematol Oncol. 2021;14(1):24. 33579329 10.1186/s13045-021-01037-x PMC7880217

[37] Huang Y, Li X, Yang L. Hydrogel encapsulation: Taking the therapy of mesenchymal stem cells and their derived secretome to the next level. Front Bioeng Biotechnol. 2022;10: 859927. 35433656 10.3389/fbioe.2022.859927 PMC9011103

[38] Yao Z, Yang Y, Kong J, Zhu Y, Li L, Chang C, Zhang C, Yin J, Chao J, Selaru FM, et al. Biostimulatory micro-fragmented nanofiber-hydrogel composite improves mesenchymal stem cell delivery and soft tissue remodeling. Small. 2022;18((36):e2202309. 35948487 10.1002/smll.202202309 PMC9994419

[39] Bai J, Ge G, Wang Q, Li W, Zheng K, Xu Y, Yang H, Pan G, Geng D. Engineering stem cell recruitment and osteoinduction via bioadhesive molecular mimics to improve osteoporotic bone-implant integration. Research. 2022;2022: 9823784. 36157511 10.34133/2022/9823784 PMC9484833

[40] Yang J, Li Y, Liu Y, Li D, Zhang L, Wang Q, Xiao Y, Zhang X. Influence of hydrogel network microstructures on mesenchymal stem cell chondrogenesis in vitro and in vivo. Acta Biomater. 2019;91:159–172. 31055122 10.1016/j.actbio.2019.04.054

[41] Ma Y, Wang X, Su T, Lu F, Chang Q, Gao J. Recent advances in macroporous hydrogels for cell behavior and tissue engineering. Gels. 2022;8(10):606. 36286107 10.3390/gels8100606 PMC9601978

[42] Schol J, Sakai D, Warita T, Nukaga T, Sako K, Wangler S, Tamagawa S, Zeiter S, Alini M, Grad S. Homing of vertebral-delivered mesenchymal stromal cells for degenerative intervertebral discs repair – An in vivo proof-of-concept study. JOR Spine. 2022;6(1):e1228. 36994461 10.1002/jsp2.1228 PMC10041374

[43] Dalir AE, Safari Z, Sadat Kachouei SS, Zabeti JR, Atashkar N, Karbalaeihasanesfahani A, Alipour M, Hashemzadeh N, Sharifi S, Dizaj SM. Cell homing strategy as a promising approach to the vitality of pulp-dentin complexes in endodontic therapy: Focus on potential biomaterials. Expert Opin Biol Ther. 2022;22(11):1405–1416. 36345819 10.1080/14712598.2022.2142466

[44] Shirke PU, Goswami H, Kumar V, Shah D, Beri S, Das S, Bellare J, Mayor S, Venkatesh VK, Seth JR, et al. “Viscotaxis”- directed migration of mesenchymal stem cells in response to loss modulus gradient. Acta Biomater. 2021;135:356–367. 34469788 10.1016/j.actbio.2021.08.039 PMC7616456

[45] Ho C, Wang C, Wu T, Kuan C, Liu Y, Wang T. Peptide-functionalized double network hydrogel with compressible shape memory effect for intervertebral disc regeneration. Bioeng Transl Med. 2022;8(2):e10447. 36925718 10.1002/btm2.10447 PMC10013763

[46] Lu C, Huang X, Yan H, Wang Y, Wang Y, Zhuo S, Wei C, Qiu H, Yang X, Zhang Y, et al. Biomimetic design of 3D fibrous mesh reinforced hydrogel replicating the form and function of the intervertebral disc. Small Structures. 2023;4(4):2200254.

[47] Hasturk O, Kaplan DL. Cell armor for protection against environmental stress: Advances, challenges and applications in micro- and nanoencapsulation of mammalian cells. Acta Biomater. 2019;95:3–31. 30481608 10.1016/j.actbio.2018.11.040 PMC6534491

[48] Matsuzawa A, Matsusaki M, Akashi M. Effectiveness of nanometer-sized extracellular matrix layer-by-layer assembled films for a cell membrane coating protecting cells from physical stress. Langmuir. 2013;29(24):7362–7368. 23092370 10.1021/la303459v

[49] Wang Z, Zhao H, Tang X, Meng T, Khutsishvili D, Xu B, Ma S. CNS organoid surpasses cell-laden microgel assembly to promote spinal cord injury repair. Research. 2022;2022: 9832128. 36061824 10.34133/2022/9832128 PMC9394056

[50] Mao AS, Özkale B, Shah NJ, Vining KH, Descombes T, Zhang L, Tringides CM, Wong SW, Shin JW, Scadden DT, et al. Programmable microencapsulation for enhanced mesenchymal stem cell persistence and immunomodulation. Proc Natl Acad Sci U S A. 2019;116(31):15392–15397. 31311862 10.1073/pnas.1819415116 PMC6681761

[51] Peng H, Chelvarajan L, Donahue R, Gottipati A, Cahall CF, Davis KA, Tripathi H, al-Darraji A, Elsawalhy E, Dobrozsi N, et al. Polymer cell surface coating enhances mesenchymal stem cell retention and cardiac protection. ACS Appl Bio Mater. 2021;4(2):1655–1667. 10.1021/acsabm.0c01473 35014513

[52] Rogan H, Ilagan F, Yang F. Comparing single cell versus pellet encapsulation of mesenchymal stem cells in three-dimensional hydrogels for cartilage regeneration. Tissue Eng Part A. 2019;25(19–20):1404–1412. 30672386 10.1089/ten.tea.2018.0289 PMC6784495

[53] An C, Liu W, Zhang Y, Pang B, Liu H, Zhang Y, Zhang H, Zhang L, Liao H, Ren C, et al. Continuous microfluidic encapsulation of single mesenchymal stem cells using alginate microgels as injectable fillers for bone regeneration. Acta Biomater. 2020;111:181–196. 32450230 10.1016/j.actbio.2020.05.024

[54] Kim H, Bae C, Kook Y-M, Koh W-G, Lee K, Park MH. Mesenchymal stem cell 3D encapsulation technologies for biomimetic microenvironment in tissue regeneration. Stem Cell Res Ther. 2019;10:51. 30732645 10.1186/s13287-018-1130-8 PMC6367797

[55] Li W, Guan T, Zhang X, Wang Z, Wang M, Zhong W, Feng H, Xing M, Kong J. The effect of layer-by-layer assembly coating on the proliferation and differentiation of neural stem cells. ACS Appl Mater Interfaces. 2015;7(7):3018–3029. 25347385 10.1021/am504456t

[56] Haddad-Mashadrizeh A, Matin MM, Shahabipour F, Ensandost S, Zomorodipour A, Bahrami AR. Effects of chitosan-glycerol phosphate hydrogel on the maintenance and homing of hAd-MSCs after xenotransplantation into the rat liver. Emergent Mater. 2022;5:519–528.

[57] Zou R, Tao J, He J, Wang C, Tan S, Xia Y, Chang X, Li R, Wang G, Zhou H, et al. PGAM5-mediated PHB2 dephosphorylation contributes to diabetic cardiomyopathy by disrupting mitochondrial quality surveillance. Research. 2022;2022:0001. 10.34133/research.0001 PMC11404314 39285950

[58] Lee MW, Luo EW-C, Silvestre-Roig C, Srinivasan Y, Akabori K, Lemnitzer P, Schmidt NW, Lai GH, Santangelo CD, Soehnlein O, et al. Apolipoprotein mimetic peptide inhibits neutrophil-driven inflammatory damage via membrane remodeling and suppression of cell lysis. ACS Nano. 2021;15(10):15930–15939. 34586780 10.1021/acsnano.1c03978 PMC8720511

[59] Sowa GA, Coelho JP, Vo NV, Pacek C, Westrick E, Kang JD. Cells from degenerative intervertebral discs demonstrate unfavorable responses to mechanical and inflammatory stimuli: A pilot study. Am J Phys Med Rehabil. 2012;91(10):846–855. 22760106 10.1097/PHM.0b013e31825f145a PMC4886479

[60] Tsai T, Cheng C, Chen C, Lai P. Mechanotransduction in intervertebral discs. J Cell Mol Med. 2014;18(12):2351–2360. 25267492 10.1111/jcmm.12377 PMC4302640

[61] Argun A, Gulyuz U, Okay O. Interfacing soft and hard materials with triple-shape-memory and self-healing functions. Macromolecules. 2018;51(7):2437–2446.

[62] Chen L, Huang T, Qiao Y, Jiang F, Lan J, Zhou Y, Yang C, Yan S, Luo K, Su L, et al. Perspective into the regulation of cell-generated forces toward stem cell migration and differentiation. J Cell Biochem. 2019;120(6):8884–8890. 30536423 10.1002/jcb.28251

[63] Kothapalli C, Mahajan G, Farrell K. Substrate stiffness induced mechanotransduction regulates temporal evolution of human fetal neural progenitor cell phenotype, differentiation, and biomechanics. Biomater Sci. 2020;8(19):5452–5464. 32996962 10.1039/d0bm01349h PMC8500671

[64] Kasper G, Dankert N, Tuischer J, Hoeft M, Gaber T, Glaeser JD, Zander D, Tschirschmann M, Thompson M, Matziolis G, et al. Mesenchymal stem cells regulate angiogenesis according to their mechanical environment. Stem Cells. 2007;25:903–910. 17218399 10.1634/stemcells.2006-0432

[65] Kusuma GD, Carthew J, Lim R, Frith JE. Effect of the microenvironment on mesenchymal stem cell paracrine signaling: Opportunities to engineer the therapeutic effect. Stem Cells Dev. 2017;26(4):617–631. 28186467 10.1089/scd.2016.0349

[66] Tytgat L, Kollert MR, Van Damme L, Thienpont H, Ottevaere H, Duda GN, Geissler S, Dubruel P, Van Vlierberghe S, Qazi TH. Evaluation of 3D printed gelatin-based scaffolds with varying pore size for MSC-based adipose tissue engineering. Macromol Biosci. 2020;20(4):1900364. 10.1002/mabi.201900364 32077631

[67] Schweizer R, Tsuji W, Gorantla VS, Marra KG, Rubin JP, Plock JA. The role of adipose-derived stem cells in breast cancer progression and metastasis. Stem Cells Int. 2015;2015:120949. 26000019 10.1155/2015/120949 PMC4427098

[68] Bartczak A, McGilvray I, Keating A. Mesenchymal stromal cell therapy to promote cardiac tissue regeneration and repair. Curr Opin Organ Transplant. 2017;22(1):86–96. 28005573 10.1097/MOT.0000000000000379

[69] Zhou QI, Yang C, Yang P. The promotional effect of mesenchymal stem cell homing on bone tissue regeneration. Curr Stem Cell Res Ther. 2017;12(5):365–376. 25670062 10.2174/1574888X10666150211160604

[70] Lee S, Karki R, Wang Y, Nguyen LN, Kalathur RC, Kanneganti T-D. AIM2 forms a complex with pyrin and ZBP1 to drive PANoptosis and host defence. Nature. 2021;597(7876):415–419. 34471287 10.1038/s41586-021-03875-8 PMC8603942

[71] Luo J, Yang Y, Wang X, Chang X, Fu S. Role of pyroptosis in intervertebral disc degeneration and its therapeutic implications. Biomolecules. 1804;2022:12(12):1804. 10.3390/biom12121804 PMC9775394 36551232

[72] Hong J, Li S, Markova DZ, Liang A, Kepler CK, Huang Y, Zhou J, Yan J, Chen W, Huang D, et al. Bromodomain-containing protein 4 inhibition alleviates matrix degradation by enhancing autophagy and suppressing NLRP3 inflammasome activity in NP cells. J Cell Physiol. 2020;235(7–8):5736–5749. 31975410 10.1002/jcp.29508

[73] Fu F, Bao R, Yao S, Zhou C, Luo H, Zhang Z, Zhang H, Li Y, Yan S, Yu H, et al. Aberrant spinal mechanical loading stress triggers intervertebral disc degeneration by inducing pyroptosis and nerve ingrowth. Sci Rep. 2021;11(1):772. 33437038 10.1038/s41598-020-80756-6 PMC7804398

[74] Wicks P, Rasouliyan L, Katic B, Nafees B, Flood E, Sasané R. The real-world patient experience of fingolimod and dimethyl fumarate for multiple sclerosis. BMC Res Notes. 2016;9(1):434. 27604188 10.1186/s13104-016-2243-8 PMC5015319

[75] Masuda K, Aota Y, Muehleman C, Imai Y, Okuma M, Thonar EJ, Andersson GB, An HS. A novel rabbit model of mild, reproducible disc degeneration by an Anulus needle puncture: Correlation between the degree of disc injury and radiological and histological appearances of disc degeneration. Spine. 2005;30(1):5–14. 15626974 10.1097/01.brs.0000148152.04401.20

[76] Pfirrmann CWA, Metzdorf A, Zanetti M, Hodler J, Boos N. Magnetic resonance classification of lumbar intervertebral disc degeneration. Spine. 2001;26(17):1873–1878. 11568697 10.1097/00007632-200109010-00011

[77] Lai A, Gansau J, Gullbrand SE, Crowley J, Cunha C, Dudli S, Engiles JB, Fusellier M, Goncalves RM, Nakashima D, et al. Development of a standardized histopathology scoring system for intervertebral disc degeneration in rat models: An initiative of the ORS spine section. JOR Spine. 2021;4(2):e1150. 34337335 10.1002/jsp2.1150 PMC8313153

