# Supplementary material for: Single-Cell Microgel Encapsulation Improves the Therapeutic Efficacy of Mesenchymal Stem Cells in Treating Intervertebral Disc Degeneration via Inhibiting Pyroptosis
Source: Research (Wash D C). 2024 Feb 16;7:0311. doi: 10.34133/research.0311 (PMC10871001; doi:10.34133/research.0311)
Supplement: Supplementary 1 — Figs. S1 to S3 [file research.0311.f1.docx]

*Supporting Information For*

**Single Cell Microgel Encapsulation Improves the Therapeutic Efficacy of Mesenchymal Stem Cells in Treating Intervertebral Disc Degeneration via Inhibiting Pyroptosis**

Guanrui Huang^1†^, Haotian Shen^1†^, Kaiwang Xu^2†^, Yifan Shen^1^, Jinjia Le^1^, Guangyu Chu^1^, Hongyuan Xing^1^, Zhiyun Feng^1*^, Yue Wang^1*^

^1^ Department of Orthopedic Surgery, The First Affiliated Hospital, Zhejiang University School of Medicine, Hangzhou 310003, China

^2^ Zhejiang University, Hangzhou 310058, China

^†^ These authors contributed equally to this work.

Corresponding author E-mail: [wangyuespine@zju.edu.cn](mailto:wangyuespine@zju.edu.cn), [fengzhiyun@zju.edu.cn](mailto:fengzhiyun@zju.edu.cn)

**Keywords**: single cell microgel, pyroptosis, disc degeneration, oxidative stress, MSC


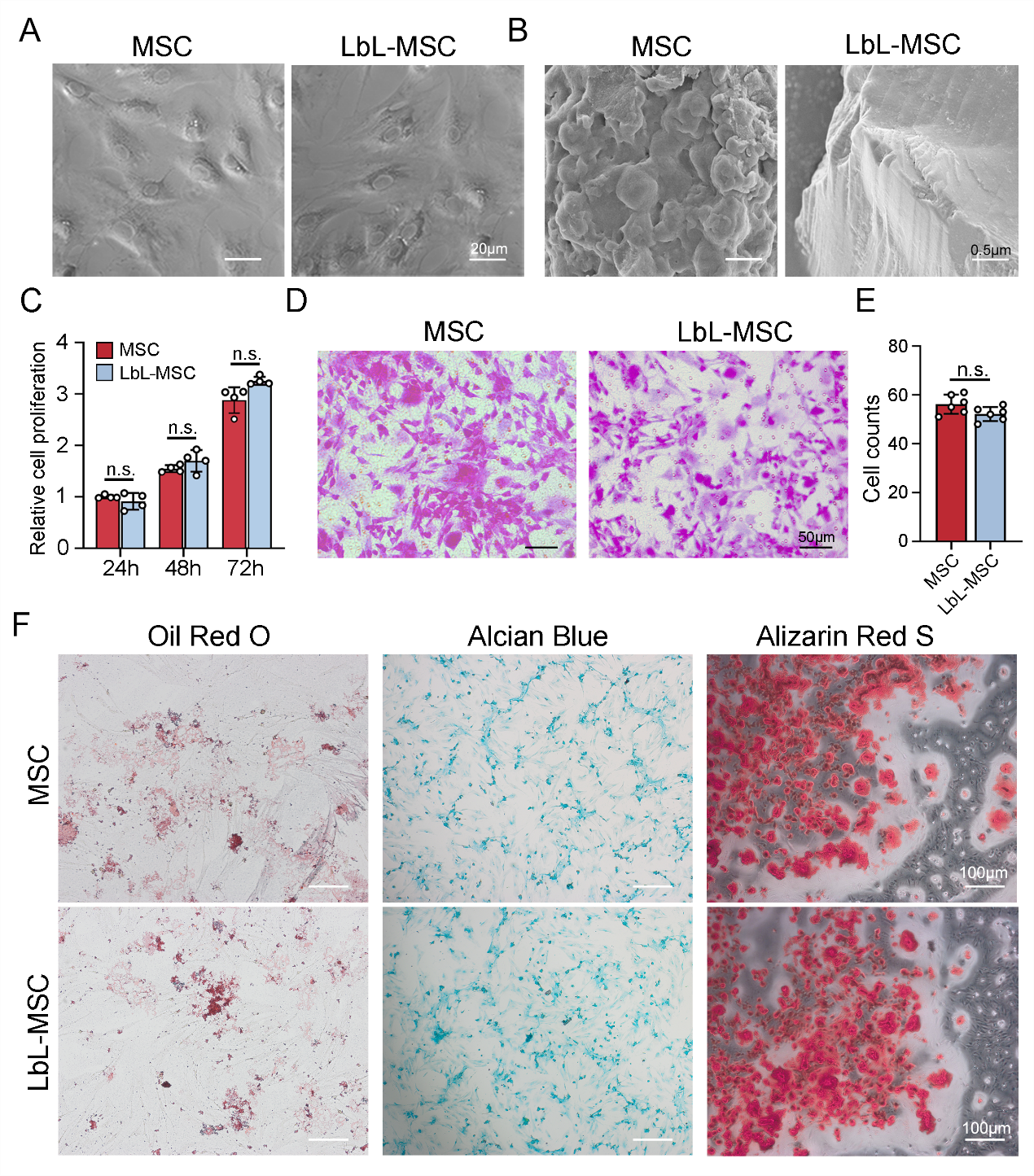


**Fig. S1 Microgel encapsulation changed the surface property but not the proliferation and function of MSC.** (A) Growth status of MSC and LbL-MSC under light microscope. (B) Change in surface property after single cell encapsulation under high magnification SEM. (C) CCK-8 analysis of cell growth ability of MSC and LbL-MSC in 24h, 48h and 72h. (D) Crystal staining of migrated cells in transwell experiment and (E) quantitative analysis. (F) Lipogenic (oil red O), chondrogenic (alcian blue) and osteogenic (alizarin red S) differentiation potential of MSC and LbL-MSC. n.s: not significant.


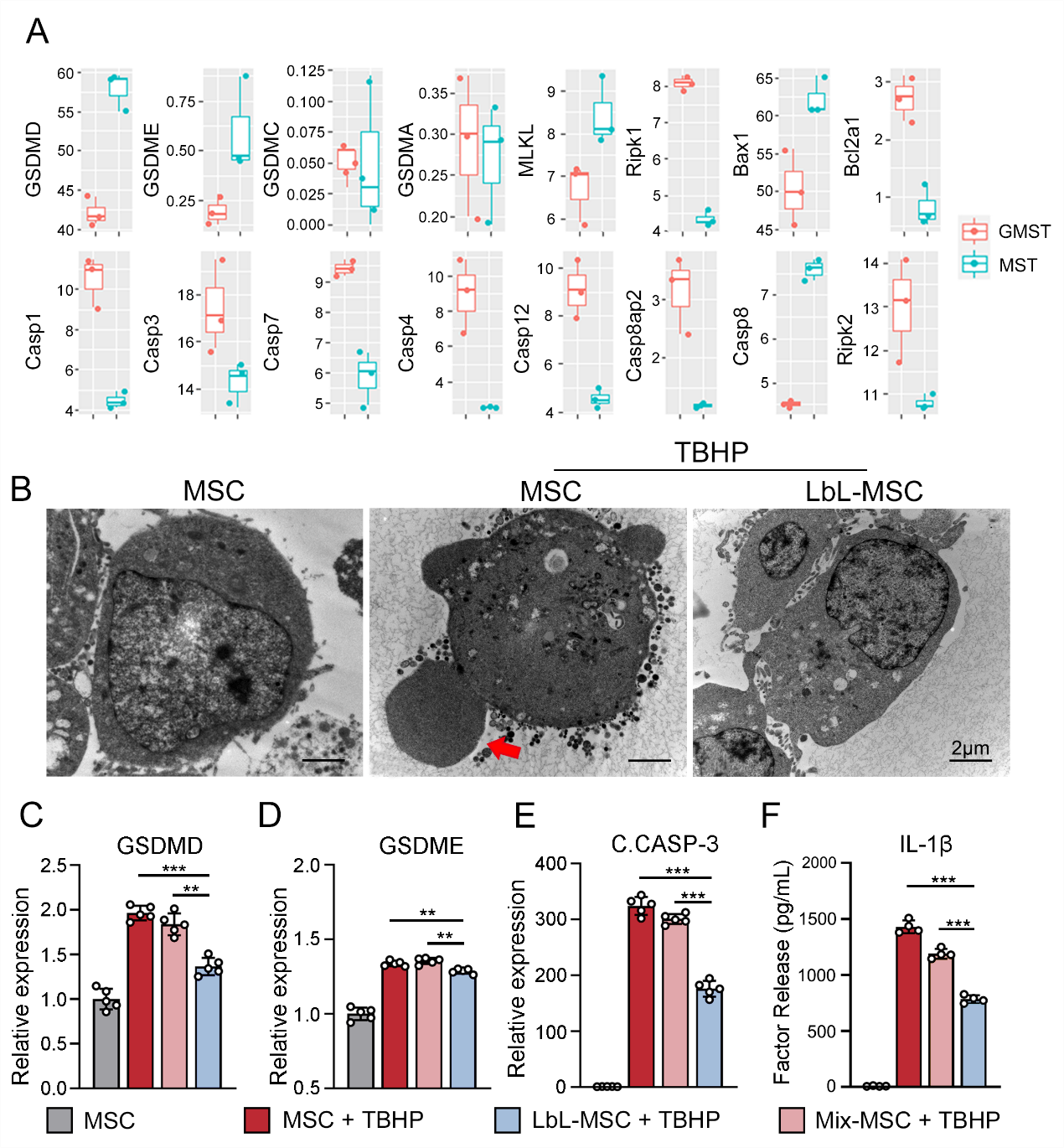


**Fig. S2 Pyroptosis protein activation in degenerated discs and pyroptosis cells. (A) Differential analysis of expression of cell death-related genes.** (B) Morphologies of MSC and LbL-MSC after TBHP treatment (TEM scanning, scale bar: 2μm). Quantification analysis of pyroptosis related proteins (C-E) and IL-1β (F). **: p<0.01; ***: p<0.001.


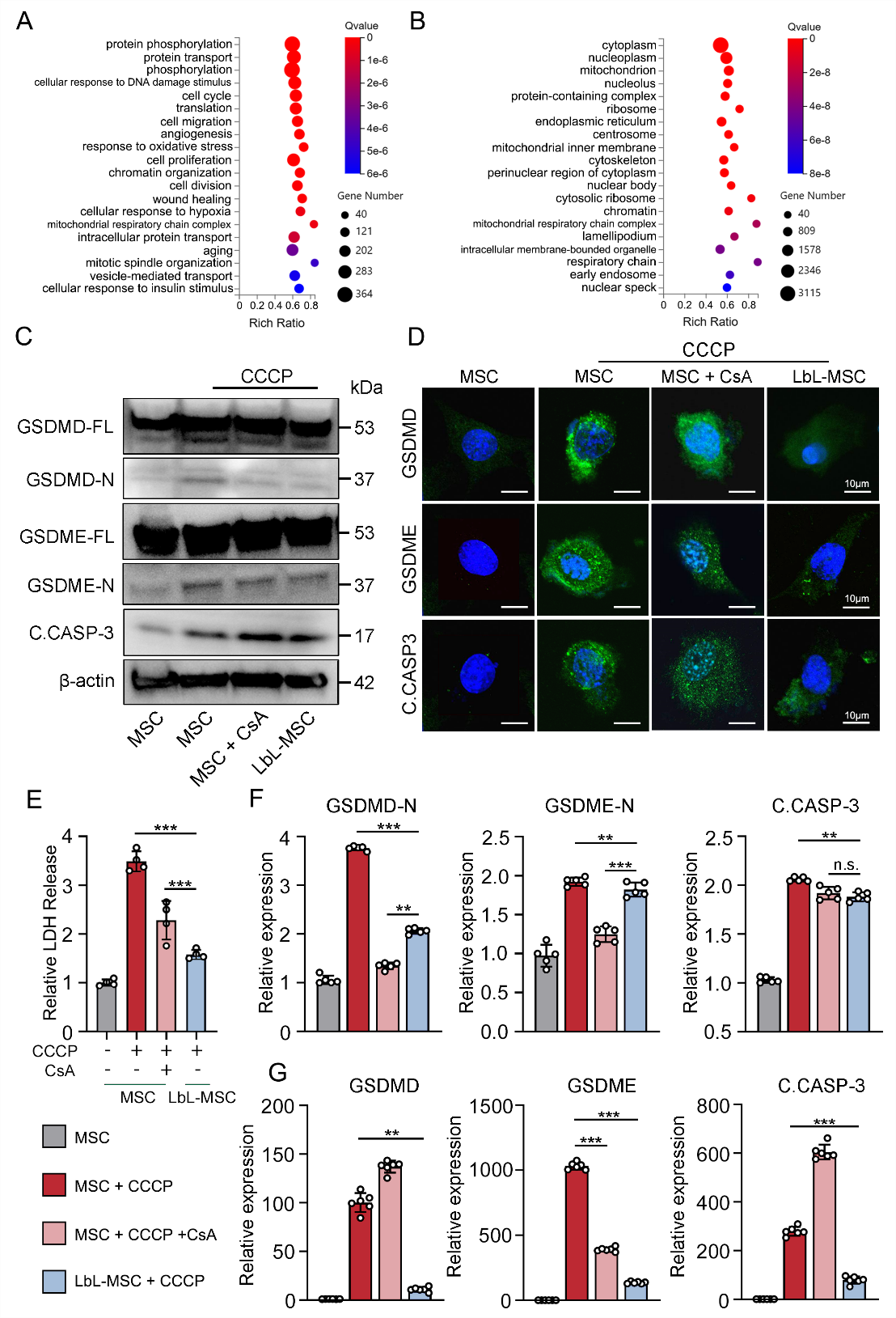


**Fig. S3 Mitochondria dysfunction activated cell pyroptosis. (A, B) GO analysis of enriched signal pathways after TBHP treatment.** (C) Western blotting and (D) immunofluorescence analysis of pyroptosis related proteins in different groups after CCCP induced mitochondria dysfunction. (E) Relative LDH release after treatment. (F) Quantification analysis of WB and (G) immunofluorescence of pyroptosis related gene in cells with mitochondria dysfunction. n.s: not significant; **: p<0.01; ***: p<0.001.
